# Risk of common psychiatric disorders, suicidal behaviours, and premature mortality following violent victimisation: A matched cohort and sibling-comparison study of 127,628 people who experienced violence in Finland and Sweden

**DOI:** 10.1371/journal.pmed.1004410

**Published:** 2024-10-18

**Authors:** Amir Sariaslan, Joonas Pitkänen, Jonas Forsman, Ralf Kuja-Halkola, Isabell Brikell, Brian M. D’Onofrio, Mikko Aaltonen, Henrik Larsson, Pekka Martikainen, Paul Lichtenstein, Seena Fazel

**Affiliations:** 1 Department of Psychiatry, University of Oxford, Warneford Hospital, Oxford, United Kingdom; 2 Helsinki Institute for Demography and Population Health, Faculty of Social Sciences, University of Helsinki, Finland; 3 Max Planck–University of Helsinki Center for Social Inequalities in Population Health, Helsinki, Finland; 4 Department of Clinical Neuroscience, Karolinska Institutet, Stockholm, Sweden; 5 Swedish National Board of Forensic Medicine, Stockholm, Sweden; 6 Department of Medical Epidemiology and Biostatistics, Karolinska Institutet, Stockholm, Sweden; 7 Department of Global Public Health and Primary Care, University of Bergen, Bergen, Norway; 8 Department of Biomedicine, Aarhus University, Aarhus, Denmark; 9 Department of Psychological and Brain Sciences, Indiana University, Bloomington, United States of America; 10 Law School, University of Eastern Finland, Joensuu, Finland; 11 Institute of Criminology and Legal Policy, Faculty of Social Sciences, University of Helsinki, Helsinki, Finland; 12 School of Medical Sciences, Örebro University, Örebro, Sweden; 13 Oxford Health NHS Foundation Trust, Oxford, United Kingdom; Massachusetts General Hospital, UNITED STATES OF AMERICA

## Abstract

**Background:**

Associations between violent victimisation and psychiatric disorders are hypothesised to be bidirectional, but the role of violent victimisation in the aetiologies of psychiatric disorders and other adverse outcomes remains unclear. We aimed to estimate associations between violent victimisation and subsequent common psychiatric disorders, suicidal behaviours, and premature mortality while accounting for unmeasured familial confounders.

**Methods and findings:**

Using nationwide registers, we identified a total of 127,628 individuals born in Finland (1987 to 2004) and Sweden (1973 to 2004) who had experienced violent victimisation, defined as either hospital admissions or secondary care outpatient visits for assault-related injuries. These were age- and sex-matched with up to 10 individuals in the general population (*n* = 1,276,215). Additionally, we matched those who had experienced violent victimisation with their unaffected siblings (*n* = 132,408). Outcomes included depression, anxiety, personality disorders, alcohol use disorders, drug use disorders, suicidal behaviours, and premature mortality. Participants were followed from the victimisation date until the date of the outcome, emigration, death, or December 31, 2020, whichever occurred first. Country-specific associations were estimated using stratified Cox regression models, which also accounted for unmeasured familial confounders via sibling comparisons. The country-specific associations were then pooled using meta-analytic models.

Among 127,628 patients (69.0% male) who had experienced violent victimisation, the median age at first violent victimisation was 21 (interquartile range: 18 to 26) years. Incidence of all outcomes was larger in those who were exposed to violent victimisation compared to population controls, ranging from 2.3 (95% confidence interval (CI) [2.2; 2.4]) per 1,000 person-years for premature mortality (compared with 0.6, 95% CI [0.6; 0.6], in controls) to 22.5 (95% CI [22.3; 22.8]) per 1,000 person-years for anxiety (compared with 7.3, 95% CI [7.3; 7.4], in controls). In adjusted models, people who had experienced violent victimisation were between 2 to 3 times as likely as their siblings to develop any of the outcomes, ranging from adjusted hazard ratio [aHR] 1.7 (95% CI [1.7; 1.8]) for depression to 3.0 (95% CI [2.9; 3.1]) for drug use disorders. Risks remained elevated 2 years post-victimisation, ranging from aHR 1.4 (95% CI [1.3; 1.5]) for depression to 2.3 (95% CI [2.2; 2.4]) for drug use disorders.

Our reliance on secondary care data likely excluded individuals with milder assault-related injuries and less severe psychiatric symptoms, thus suggesting that our estimates may be conservative. Another limitation is the possibility of residual genetic confounding, as full siblings share on average about half of their co-segregating genes. However, the associations remained robust even after adjusting for both measured and unmeasured familial confounders.

**Conclusions:**

In this longitudinal cross-national cohort study, we observed that those who had experienced violent victimisation were at least twice as likely as their unaffected siblings to develop common psychiatric disorders (i.e., depression, anxiety, personality disorder, and alcohol and drug use disorders), engage in suicidal behaviours, and to die prematurely. Importantly, these risk elevations remained 2 years after the first victimisation event. Improving clinical assessment, management, and aftercare psychosocial support could therefore potentially reduce rates of common psychiatric disorders, suicidality, and premature mortality in individuals experiencing violent victimisation.

## Abstract 2

Amir Sariaslan and team examine whether severe forms of violence contribute to the development of psychiatric disorders using a matched cohort and sibling-comparison study design.

## Author summary

**Why was this study done?:**

- Violent victimisation risk is at least doubled in people diagnosed with psychiatric disorders, and its prevention is an important public mental health challenge.
- The association between violent victimisation and psychiatric disorders is considered to be bidirectional, but few studies have examined whether experiencing severe forms of violence contribute to the development of psychiatric disorders.
- As previous work has reported mixed findings, there is a need to re-examine this question using large-scale data with rigorous research designs that can account for key confounders.

**What did the researchers do and find?:**

- We used nationwide register data from Finland and Sweden that covered a total of 4.2 million individuals born between 1973 and 2004.
- This approach allowed us to identify more than 127,000 individuals who had been exposed to violent victimisation severe enough to require specialist medical care.
- We compared individuals who had been exposed to violent victimisation with their siblings without violent victimisation to account for shared factors (e.g., genetic risks and childhood environmental influences).
- We found that those who had been experienced violent victimisation were 2 to 3 times more likely than their unaffected siblings to develop common psychiatric disorders (i.e., depression, anxiety, personality disorder, and alcohol and drug use disorders), engage in suicidal behaviours, and to die prematurely.
- These risks remained elevated for more than 2 years post-victimisation.

**What do these findings mean?:**

- Violent victimisation is a key risk factor for subsequent common psychiatric disorders, suicidal behaviours, and premature mortality, with risks that persist over years.
- Personalising clinical assessment across emergency departments and general hospitals could improve management of individuals experiencing violent victimisation.
- Presentations to medical services that involve victimisation provide an opportunity to assess, prevent and treat emerging psychiatric symptoms and conditions.
- While the sibling-comparison offers a powerful approach to account for unmeasured familial confounders, it does not account for non-shared familial confounders, and will underestimate causal effects in the presence of misclassification bias, shared mediators, and sibling carryover effects.

## Introduction

People diagnosed with psychiatric disorders are at least twice as likely as the general population to be violently victimised [1–3]. This finding has been consistently replicated across several victimisation outcomes, including self-reports [1], police-reported events [4,5], assaults requiring medical care [6,7], and homicidal deaths [8]. Although it is commonly hypothesised that associations between common psychiatric disorders and violent victimisation are bidirectional in nature, few studies have rigorously examined whether exposure to violent victimisation contributes to the development of common psychiatric disorders and suicidal behaviours.

Two key methodological limitations are present in previous work that has considered violent victimisation as a risk factor for psychiatric disorders. First, violent victimisation has been combined with several broader indices of adverse childhood experiences, bullying or negative/stressful life events [2,9,10]. While this has the benefit of simultaneously considering multiple types of adversities, they do not permit the evaluation of the specific contributions of severe violent victimisation to aetiologies of psychiatric disorders, suicidal behaviours, and premature mortality.

Second, twin studies have consistently demonstrated that victimisation is moderately heritable, with genetic factors accounting for around 20% to 40% of the variance [11,12]. These findings are expected, as victimisation is also associated with a vast array of risk factors that are at least moderately heritable, including low cognitive abilities [13], personality traits [14], and violent perpetration [15]. As these risk factors are implicated in the aetiologies of common psychiatric disorders, suicidal behaviours, and premature mortality, unmeasured genetic confounding could account for at least a portion of the associations. The few genetically informative studies that have examined the long-term associations between violent victimisation and subsequent common psychiatric disorders have reported conflicting findings, in part due to limited statistical power.

A recent study [16] that considered unmeasured familial confounding in Sweden reported that people who had experienced an assault were 3 times as likely as their unaffected siblings to develop any psychiatric disorders within the year following the assault, and twice as likely thereafter. However, follow-up of the study ended in 2013, many relevant victimisation exposures (i.e., assaults by strangulation and poisoning) were not included, sex-specific associations were not investigated for specific outcomes, and the findings have not been externally replicated.

To address these evidence gaps, we examined the extent to which exposure to severe forms of violent victimisation was associated with subsequent risks of common psychiatric disorders, suicidal behaviours, and premature mortality by pooling nationwide register data from Finland and Sweden. In complementary analyses, we also tested for effect moderation by preexisting psychiatric disorders. We were further able to account for unmeasured familial confounders, such as shared genetic and early environmental risk factors, by comparing risks of the outcomes between siblings who were differentially exposed to violent victimisation. We complemented the sibling-comparison design by additionally accounting for measured confounders that varied between the siblings over time, such as being exposed to single motherhood and low family income at birth.

## Methods

### National registers

A unique personal identification number is assigned either at birth or upon immigration to all Finnish and Swedish residents, thus enabling accurate linkage across various national social and health registers [17,18]. We were granted access to pseudonymised administrative population data by the Ethics Board of Statistics Finland (TK-53-1490-18), the Finnish Institute of Health and Welfare (THL/2180/14.02.00/2020), and the Swedish Ethical Review Authority (Dnr 2020–06540; Dnr 2022-06204-02). Informed consent is neither mandated by Finnish nor Swedish law for research involving national registers with pseudonymised identifiers. The study followed the STROBE reporting guidelines (**Checklist A in S1 Supporting Information**), but did not have a prospective analysis plan.

The Care Register for Health Care, maintained by the Finnish Institute for Health and Welfare (THL), and the National Patient Register, maintained by the Swedish National Board for Health and Welfare, contain information on inpatient hospitalisation episodes (Finland: 1969–2020; Sweden: 1973–2020) and specialist outpatient care (Finland: 1998–2020; Sweden: 2001–2020). Diagnoses were classified according to the Finnish and Swedish versions of the International Classification of Diseases (ICD) from the 8th to 10th revisions. Data on mortality dates, including the primary and contributing causes of death, were extracted from the Causes of Death Registers of each country, which utilised the same ICD classifications as the hospital data. Data on sociodemographic factors, including emigration dates, were retrieved from a series of population administrative registers maintained by Statistics Finland and Statistics Sweden.

Using these registers, we identified 1,090,641 individuals born in Finland between 1987 and 2004 and 3,269,545 individuals born in Sweden between 1973 and 2004. Individuals who could not be linked to their biological parents (n_Finland_ = 15,406; n_Sweden_ = 42,340) or who lacked data on parental sociodemographic factors (n_Finland_ = 1,900; n_Sweden_ = 72,468) were excluded. The resulting analytical samples comprised 98.4% (*n* = 1,073,335) of the targeted Finnish sample and 96.5% (*n* = 3,154,737) of the Swedish sample, yielding a pooled sample of 4,228,072 individuals.

### Violent victimisation

Violent victimisation was defined as an inpatient care episode or secondary care outpatient visit associated with a diagnosis of an injury purposefully inflicted by another person (ICD-codes are provided in **Table A** in **S1 Supporting information**; see also **Text A** in **S1 Supporting information**).

### Common psychiatric disorder, suicidal behaviours, and premature mortality

The following common psychiatric disorders were investigated as outcomes: depression, anxiety, personality disorders, alcohol use disorders, and drug use disorders. The measures of alcohol and drug use disorders also included deaths attributable to these conditions. We additionally investigated suicidal behaviours, encompassing both nonfatal self-harm and completed suicide, and also examined premature mortality, defined consistent with the literature as any death prior to the age of 65 years [19]. We further included preexisting measures of the psychiatric disorders and self-harm.

### Control groups

We matched each index person experiencing violent victimisation with up to 10 individuals from the general population without such experiences throughout the follow-up by sex and birth year. These control individuals were required to be alive and residing in Finland or Sweden at the time of the first violent victimisation event of the index person. We also matched each index person with their biological full-siblings who had not experienced violent victimisation to account for unmeasured shared familial confounding. In these analyses, we included all siblings and adjusted for sex and birth year differences using statistical covariates. If multiple siblings in a given family had experienced violent victimisation, we matched them with all their unaffected co-siblings in separate strata [19]. To account for the fact that the same individuals could serve as controls across multiple strata, we specified all sibling models to estimate individual-level cluster-robust standard errors.

By comparing outcome rates between differentially exposed siblings, we accounted for time invariant and unmeasured familial confounders shared between the siblings, including an average of half of their co-segregating genes and their shared childhood environments [20]. The latter factors were accounted for by the design, and therefore not directly measured. The extent to which the sibling comparisons were attenuated from the population estimates indicated the influence of these unmeasured familial confounders on the studied associations, assuming no misclassification of the exposure and outcome variables.

The starting points for both index persons and control groups were defined as the date of the first violent victimisation event. Participants were censored if they migrated, died, experienced the outcome of interest, or reached the endpoint of the study on December 31, 2020.

### Analytical strategy

In each country, we fitted separate stratified Cox proportional hazards analyses to estimate adjusted hazard ratios (aHRs) for the risk of common psychiatric disorders, suicidal behaviours, and premature mortality among individuals who had experienced violent victimisation compared with matched individuals without such an experience, with time in years since the first victimisation event (or equivalent date for the matched individuals) as the underlying time scale. As each index person who experienced violent victimisation and their corresponding control individuals were assigned to a separate stratum, the models estimated varying baseline hazards across these strata, indicating that comparisons were made within each stratum.

We fitted 3 models that gradually accounted for a larger set of potential confounders. The “Crude” model only accounted for the matched characteristics (e.g., sex and birth year). The “Adjusted” model, additionally accounted for the following measured confounders (**Table B in S1 Supporting Information**): birth order, immigrant background, parental characteristics measured at birth (low family income, single mother, parental psychiatric disorders, and violent criminality) in addition to any psychiatric disorders and self-harm that had occurred prior to the victimisation event (or the equivalent period for the controls). The “Sibling-comparison” model further accounted for unmeasured familial confounders by fitting the adjusted model on a separate sample that included index persons who had experienced violent victimisation and their biological full-siblings who did not.

To assess the moderating role of preexisting psychiatric disorders and self-harm behaviours on the associations under investigation, we added an interaction term to the sibling-comparison models. We used an aggregated binary measure of all preexisting conditions to reduce the number of hypothesis tests. To aid interpretation, we estimated linear combinations of the model parameters to assess the differential associations of preexisting conditions in the presence and absence of violent victimisation. Interaction terms that did not reach statistical significance (*P* > 0.05) were omitted from the models.

Consistent with prior studies [21,22], we fitted inverse variance-weighted fixed-effects meta-analysis models to the resulting regression coefficients and standard errors from the country-specific models [23], which allowed us to pool the associations across both countries by weighting the magnitude of the associations by their sample sizes.

In complementary sensitivity analyses, we examined an alternative exposure measure, which included individuals who were registered as victims of violent offences (e.g., attempted homicide, assault, robbery, unlawful threats, or sexual offences) in Finnish police reports between 2008 and 2020 and who did not have corresponding hospital episode. Additional sensitivity analyses are described elsewhere (**Text B** in **S1 Supporting Information**).

## Results

A total of 127,628 individuals had experienced violent victimisation in Finland and Sweden at a median age of 21 years, with males accounting for 69% of the group (**Table 1; Tables C and D in S1 Supporting information**). When compared to age and sex-matched controls (*n* = 1,276,215), those who experienced violent victimisation were more likely to come from backgrounds characterised by low family income, a single mother household, and parents with a history of psychiatric disorders and violent crime convictions at offspring birth (**Table 1**).

**Table 1:** Baseline demographic characteristics.

	General population controls	Individuals exposed to violent victimisation	Unaffected sibling controls	Individuals exposed to violent victimisation with unaffected siblings
**Total, *n***	1,276,215	127,628	132,408	85,825
**Median age at victimisation (IQR) years**	-	21 (18–26)	-	21 (18–25)
**Sex, *n* (%)**				
Female	395,990 (31.0%)	39,599 (31.0%)	66,048 (49.9%)	25,714 (30.0%)
Male	880,225 (69.0%)	88,029 (69.0%)	66,360 (50.1%)	60,111 (70.0%)
**Birth cohorts, *n* (%)**				
1973–1974	51,940 (4.1%)	5,194 (4.1%)	3,446 (2.6%)	1,990 (2.3%)
1975–1979	134,290 (10.5%)	13,429 (10.5%)	13,021 (9.8%)	7,820 (9.1%)
1980–1984	179,270 (14.0%)	17,927 (14.0%)	19,316 (14.6%)	12,495 (14.6%)
1985–1989	307,062 (24.1%)	30,712 (24.1%)	29,948 (22.6%)	21,530 (25.1%)
1990–1994	327,253 (25.6%)	32,726 (25.6%)	34,519 (26.1%)	23,700 (27.6%)
1995–1999	184,320 (14.4%)	18,432 (14.4%)	20,740 (15.7%)	13,070 (15.2%)
2000–2004	92,080 (7.2%)	9,208 (7.2%)	11,418 (8.6%)	5,220 (6.1%)
**Birth order, *n* (%)**				
1st	773,757 (60.6%)	75,172 (58.9%)	65,569 (49.5%)	47,213 (55.0%)
2nd	366,160 (28.7%)	36,619 (28.7%)	40,939 (30.9%)	26,881 (31.3%)
3rd	101,593 (8.0%)	11,537 (9.0%)	16,676 (12.6%)	8,456 (9.9%)
4th or higher	34,705 (2.7%)	4,300 (3.4%)	9,224 (7.0%)	3,275 (3.8%)
**Immigration background, *n* (%)**	197,297 (15.5%)	26,495 (20.8%)	26,658 (20.1%)	16,571 (19.3%)
**Mother single at offspring birth, *n* (%)**	520,563 (40.8%)	63,189 (49.5%)	53,410 (40.3%)	37,770 (44.0%)
**Family income in the bottom decile at offspring birth, *n* (%)**	119,059 (9.3%)	17,018 (13.3%)	13,165 (9.9%)	9,332 (10.9%)
**Parental history of psychiatric disorders, *n* (%)**	54,486 (4.3%)	11,381 (8.9%)	9,594 (7.2%)	6,048 (7.0%)
**Parental history of violent criminality, *n* (%)**	67,265 (5.3%)	16,476 (12.9%)	14,644 (11.1%)	9,439 (11.0%)
**Pre-victimisation psychiatric disorders and self-harm, *n* (%)**				
Depression	42,363 (3.3%)	13,327 (10.4%)	6,833 (5.2%)	8,300 (9.7%)
Anxiety	46,378 (3.6%)	14,679 (11.5%)	7,113 (5.4%)	9,009 (10.5%)
Personality disorder	7,865 (0.6%)	4,419 (3.5%)	1,482 (1.1%)	2,582 (3.0%)
Alcohol use disorder	23,856 (1.9%)	12,125 (9.5%)	4,346 (3.3%)	7,458 (8.7%)
Drug use disorder	10,599 (0.8%)	9,497 (7.4%)	2,268 (1.7%)	5,502 (6.4%)
Other psychiatric disorders	68,656 (5.4%)	11,928 (9.3%)	8,730 (6.6%)	7,494 (8.7%)
Self-harm	23,395 (1.8%)	10,483 (8.2%)	4,095 (3.1%)	6,382 (7.4%)

Notes: IQR refers to interquartile range.

**Fig 1:**
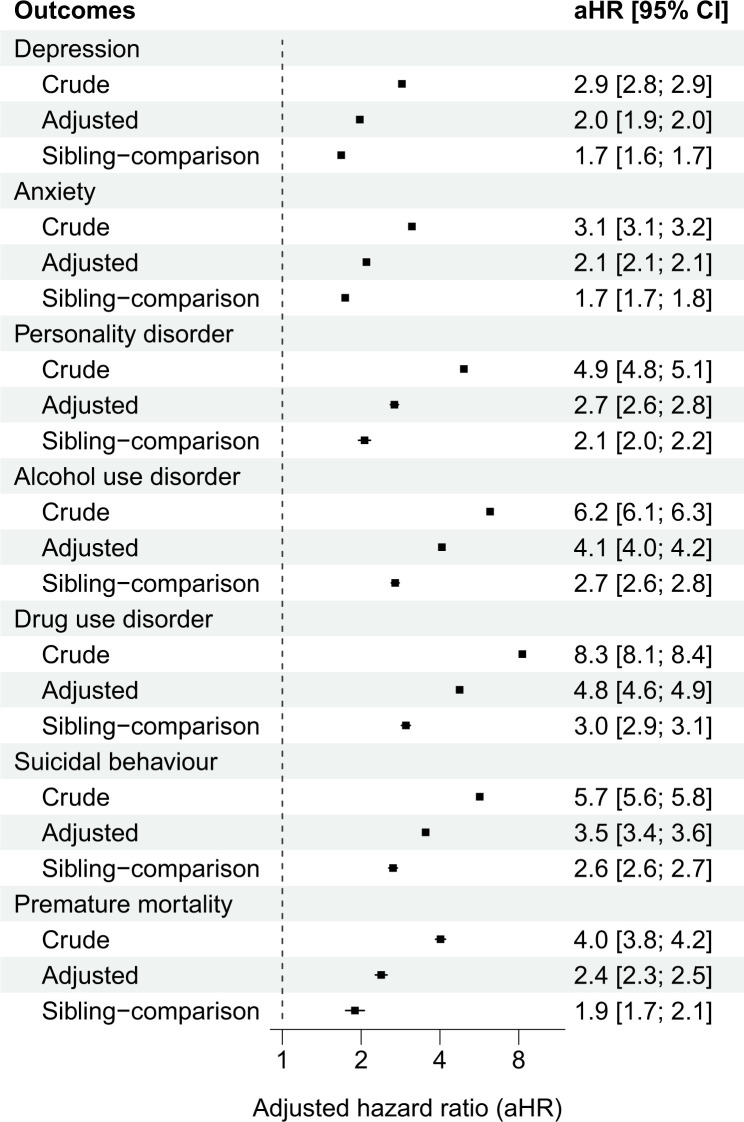
Population-wide and within-family associations between violent victimisation and subsequent common psychiatric disorders, suicidal behaviours, and premature mortality in Finland and Sweden. Notes: The “Crude” model adjusted for sex and birth year. The “Adjusted” model additionally accounted for birth order, parental immigrant background, low family income at offspring birth, single mother at offspring birth, parental psychiatric, and violent crime histories at offspring birth, as well as pre-victimisation psychiatric disorders, alcohol use disorders, drug use disorders, and self-harm. The “Sibling-comparison” model refers to within-family estimates comparing biological full-siblings differentially exposed to violent victimisation and is adjusted for all time-invariant unmeasured familial confounders shared between the siblings as well as the following measured confounders that vary within families: sex, birth year, birth order, and parental characteristics at birth (low family income, single mother, psychiatric history, and violent crime history) as well as any preexisting psychiatric disorders and self-harm events. CI refers to confidence intervals.

The median follow-up time was 9.5 years across all outcomes (**Table 2; Table E in S1 Supporting information**). Individuals who had experienced violent victimisation had considerably elevated rates of all outcomes compared to the population controls (**Table 2**). Individuals who had experienced violent victimisation were at least 3 times as likely as the population controls, matched for sex and birth year, to have experienced any of the outcomes (**Fig 1**), ranging from a 3-fold elevated risk for being diagnosed with depression to over an 8-fold risk increase of being diagnosed with a drug use disorder. Although these estimates were attenuated as we gradually accounted for measured confounders and unmeasured familial confounders, they remained being associated with a 2- to 3-fold increased risk of the outcomes in the sibling-comparison models (**Fig 1**).

**Table 2:** Person-time at risk, number of individuals with the outcomes, and incident rates per 1,000 person-years for common psychiatric disorders, suicidal behaviours, and premature mortality stratified across individuals exposed to violent victimisation.

	Exposure to violent victimisation	Population size, *n*	Person-years at risk, years (mean)	Number of patients, *n*	Incidence rate per 1,000 person-years [95% CI]
**Depression**					
	Unexposed	1,276,215	11,911,416 (9.3)	72,644	6.1 [6.1; 6.1]
	Exposed	127,628	1,102,703 (8.6)	19,125	17.3 [17.1; 17.6]
**Anxiety**					
	Unexposed	1,276,215	11,847,812 (9.3)	86,839	7.3 [7.3; 7.4]
	Exposed	127,628	1,078,814 (8.5)	24,327	22.5 [22.3; 22.8]
**Personality disorder**					
	Unexposed	1,276,215	12,241,293 (9.6)	16,679	1.4 [1.3; 1.4]
	Exposed	127,628	1,172,997 (9.2)	7,836	6.7 [6.5; 6.8]
**Alcohol use disorder**					
	Unexposed	1,276,215	12,158,990 (9.5)	28,012	2.3 [2.3; 2.3]
	Exposed	127,628	1,113,927 (8.7)	15,985	14.4 [14.1; 14.6]
**Drug use disorder**					
	Unexposed	1,276,215	12,214,125 (9.6)	21,972	1.8 [1.8; 1.8]
	Exposed	127,628	1,122,966 (8.8)	16,758	14.9 [14.7; 15.2]
**Suicidal behaviours**					
	Unexposed	1,276,215	12,180,383 (9.5)	24,502	2.0 [2.0; 2.0]
	Exposed	127,628	1,138,494 (8.9)	12,961	11.4 [11.2; 11.6]
**Premature mortality**					
	Unexposed	1,276,215	12,341,233 (9.7)	7,089	0.6 [0.6; 0.6]
	Exposed	127,628	1,223,737 (9.6)	2,806	2.3 [2.2; 2.4]

Notes: CI refers to confidence intervals.

There was little evidence the presented associations varied systematically across the included countries, birth cohorts, or age at victimisation categories (**Figures A–C in S1 Supporting information**). However, the association between violent victimisation and suicidal behaviours was more pronounced in Finland than in Sweden (**Figure A in S1 Supporting information**). Furthermore, the associations tended to be stronger among females than males across all outcomes except for premature mortality (**Figure D in S1 Supporting information;** sex-specific absolute risks are presented in **Table F in S1 Supporting information**).

**Fig 2:**
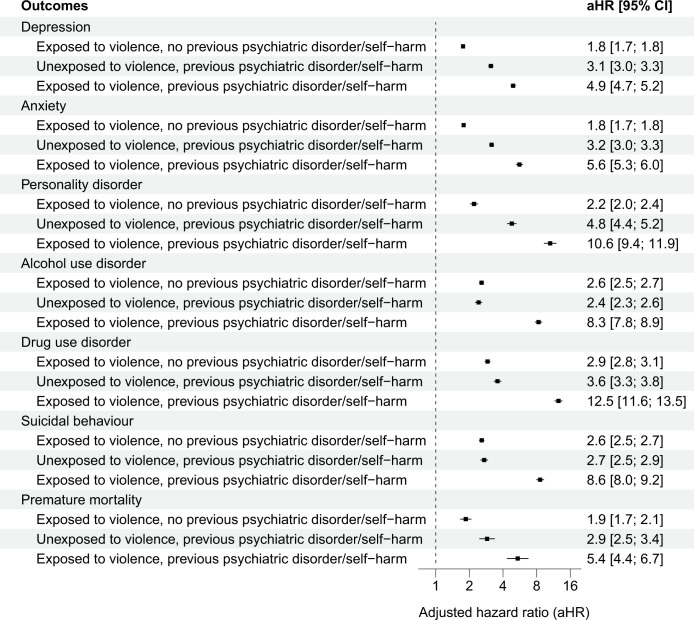
Within-family associations between violent victimisation and subsequent common psychiatric disorders, suicidal behaviours, and premature mortality among Finnish and Swedish siblings with and without previous psychiatric disorders or self-harm. Notes: The reference group included co-siblings who were unexposed to violence and had no preexisting psychiatric disorders or self-harm events. The estimates refer to within-family associations comparing biological full-siblings differentially exposed to violent victimisation and is adjusted for all time-invariant unmeasured familial confounders shared between the siblings as well as the following measured confounders that vary within families: sex, birth year, birth order, and parental characteristics at birth (low family income, single mother, psychiatric history, and violent crime history) as well as any preexisting psychiatric disorders and self-harm events. CI refers to confidence intervals.

We found some evidence in support of a moderating effect of preexisting psychiatric disorders and self-harm (interaction terms presented in **Table G in S1 Supporting information**). For depression, however, this effect modification appeared to be negative, which suggests that the combined contributions of preexisting conditions and violent victimisation were slightly weaker than the sum of their individual contributions. Conversely, for alcohol and drug use disorders as well as suicidal behaviours, the effect modification was positive, indicating that the joint contributions of preexisting conditions and violent victimisation appeared to be greater than the sum of their individual contributions. By examining the 4 exposure groups (i.e., siblings with and without preexisting psychiatric disorders, self-harm, and violent victimisation experiences), we found negligible differences from the primary findings when restricting the analyses to those without preexisting psychiatric disorders and self-harm episodes (**Figs 1 and 2**). Among those with preexisting conditions, however, we found that violent victimisation was associated with considerably higher rates of all outcomes (**Fig 2**).

**Fig 3:**
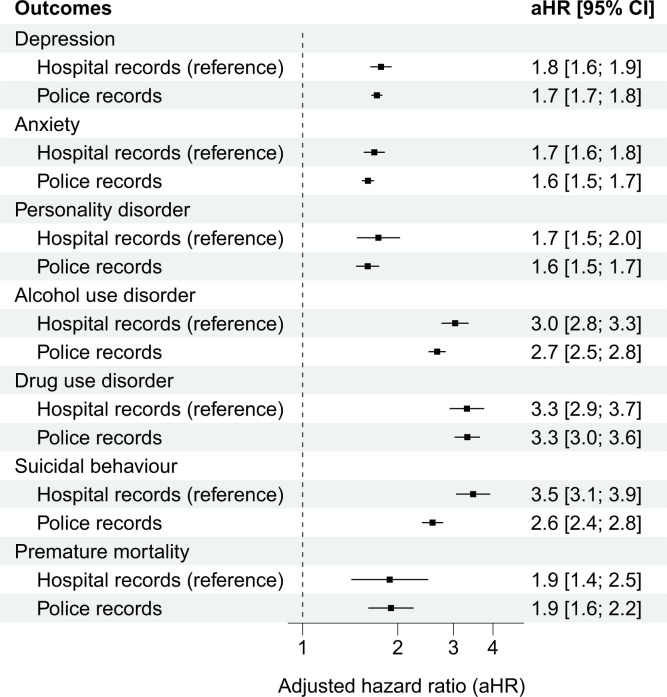
Within-family associations between specific types of violent victimisation (e.g., hospital or police records) and subsequent common psychiatric disorders, suicidal behaviours, and premature mortality among Finnish siblings. Notes: The analyses of police recorded violent victimisation were based on 43,487 Finnish individuals born between 1987 and 2004 who were registered by law enforcement as being victims of violent crimes but had not received specialised medical care for their injuries. They were matched with their unexposed siblings born during the same period (*n* = 68,107). For improved comparability, the reference estimates for hospital records were solely based on Finnish siblings. The estimates refer to within-family associations comparing biological full-siblings differentially exposed to violent victimisation and is adjusted for all time-invariant unmeasured familial confounders shared between the siblings as well as the following measured confounders that vary within families: sex, birth year, birth order, and parental characteristics at birth (low family income, single mother, psychiatric history, and violent crime history) as well as any preexisting psychiatric disorders and self-harm events. CI refers to confidence intervals.

In Finland, where we additionally investigated a different method to ascertain victimisation, associations between hospital or police recorded victimisation, and outcomes were similar in magnitude (**Fig 3**). The only exception involved suicidal behaviours; assaults requiring medical attention were associated with elevated risks than assaults that had only been reported to the police. Starting the follow-up period up to 2 years after the first victimisation date had a marginal impact on the sibling-comparison estimates across both countries, thus suggesting that the associations remained over a longer time period (**Figure E in S1 Supporting information**). We found commensurate results when adjusting for additional indicators of low parental socioeconomic status (SES) (**Figure F in S1 Supporting information**), requiring a minimum of 2 diagnoses of the psychiatric disorders on separate occasions (**Figure G in S1 Supporting information**), considering victimisation events that occurred after 2005 (**Figure H in S1 Supporting information**), excluding individuals with preexisting psychiatric disorders (**Figure I in S1 Supporting information**), and examining stress-related disorders as outcome (**Figure J in S1 Supporting information**). Low parental SES did not moderate the associations between violent victimisation and the examined outcomes (“Additional sensitivity tests” in **S1 Supporting information**).

## Discussion

In this cross-national cohort study of 127,628 individuals who were exposed to violent victimisation, each matched with up to 10 general population controls of the same age and sex, we investigated associations between violent victimisation and subsequent risks of common psychiatric disorders, suicidal behaviours, and premature death. We report 5 key findings.

First, we found that people who experienced violent victimisation had considerably elevated rates of common psychiatric disorders, suicidal behaviours, and premature mortality compared to age and sex matched individuals in the general population (median outcome rates: 17.3 versus 6.1 events per 1,000 person-years). Expressed as relative risks, those who had experienced violent victimisation had at least a 3-fold increased risk of the examined outcomes compared to the general population controls. Following more rigorous adjustments for an extensive set of measured confounders that captured sociodemographic background factors and preexisting diagnoses of psychiatric disorders and self-harm, the associations were attenuated but remained strong. These findings are consistent with studies that have reported elevated rates of psychiatric disorders and related behavioural outcomes in people who have been exposed to various measures of victimisation, including peer victimisation [24,25,10] and intimate partner violence [26–28].

Second, our estimates were further attenuated once we had accounted for unmeasured familial confounders, but they still indicated that violent victimisation was associated with least a 2-fold elevated risk of all outcomes. We tested for unmeasured familial confounding by comparing outcome rates in people who had experienced violent victimisation with their siblings without such experiences. This approach was especially informative for the associations with alcohol use disorders, drug use disorders, and suicidal behaviours, which were attenuated from a 4- to 5-fold risk increase in the adjusted population-wide models to 2- to 3-fold risk increase in the sibling-comparison models. These findings add to previous work that has shown the importance of accounting for unmeasured familial confounders when assessing mental health outcomes following violent victimisation [16,29,30]. In addition, it suggests that previously reported associations [31] without such adjustments are likely overestimated.

Third, we found that violent victimisation had a stronger association with psychiatric disorders and suicidal behaviours in females than in males. However, we found no evidence that sex moderated the corresponding associations with premature mortality. While previous research has generally suggested that females experience more severe mental health, alcohol and drug misuse outcomes following violent victimisation [31], these are based on small sample sizes and inadequate adjustments for confounders [28]. One previous Swedish nationwide study [16] reported a slightly larger relative risk of being diagnosed with any psychiatric disorder following assault victimisation among females compared with males, but that study did not consider specific diagnostic categories as reported in the present study. By investigating 2 large nationwide samples and adjusting for an extensive set of both measured and unmeasured confounders, our findings therefore help clarify the sex-specific associations between violent victimisation and subsequent risks for multiple psychiatric disorders, suicidal behaviours, and premature mortality. Future research could investigate whether these observed sex differences are further modified by the nature of the victim–perpetrator relationship, as well as other forms of non-physical violence, including emotional abuse, coercive control, economic abuse, and cyberbullying, which are potentially important risk factors for adverse outcomes.

Fourth, individuals with a history of psychiatric disorders and self-harm, but no experience of violent victimisation, had a 3- to 4-fold increased risk of experiencing the examined outcomes compared to their siblings lacking such experiences. However, those who had experienced violent victimisation, in addition to having preexisting conditions, were around 5 to 10 times as likely as their siblings without any psychiatric or victimisation history to experience the outcomes. These findings suggest that the bidirectional relationship [6,5,16] between violent victimisation and psychiatric disorders and related behaviours may potentially follow an amplification model in which the exposures and outcomes mutually reinforce each other over time.

Fifth, we were able to replicate our findings by using a different objective method for measuring violent victimisation, which was police-reported violent victimisation. This approach allowed us to identify over twice as many people experiencing violent victimisation, and this replication suggests that our main findings may be generalisable to violent victimisation that does not lead to medical attention.

Family-based studies that have examined associations between violent victimisation and subsequent risks for psychiatric disorders and related behavioural outcomes have reported conflicting findings to date [16,29,30]. In addition to updated data and external replication, the present study examined several important violent victimisation exposures that were not investigated in the prior study, including assaults by strangulation and poisoning, as well as sex-specific associations between violent victimisation and a range of psychiatric disorders and suicidal behaviours. Moreover, our findings are also consistent with a recent within-individual study [22] conducted using nationwide registers that reported a 2- to 3-fold increased odds of self-harm occurring in the week following violent victimisation compared to earlier periods within the same person. The present study uses a more comprehensive measure of violent victimisation and investigates longer-term impact on multiple psychiatric outcomes. We also investigated premature mortality, which is to our knowledge a novel outcome of increasing importance to public mental health.

Strengths include the use of high-quality nationwide register data with over 127,000 people who had been assaulted to the extent that they required specialist medical care, and around 43,500 individuals who had been identified by the police as victims of a violent offence. The registers allowed us to match each of these individuals with general population controls of the same age and sex, and also with their siblings without violent victimisation experiences to rigorously account for their shared and time-invariant unmeasured familial confounders.

There are some limitations. First, our approach to measuring violent victimisation, common psychiatric disorders, and suicidal behaviours was based on diagnosis with these conditions and injuries in secondary care settings, reflecting the more severe presentation of these. While alternative methods, such as self-reporting and proxy-reporting, can be employed to assess less severe forms of violent victimisation and psychiatric disorders, they have important methodological limitations, including non-response, reporting biases of sensitive information and longitudinal attrition. Moreover, as exemplified by the prior inconsistent findings, collecting self-reported data from a sufficiently large cohort of relatives to accurately determine associations between rare exposures and outcomes remains a significant challenge.

Second, while healthcare registers in Finland and Sweden provide accurate admission and discharge dates, these dates do not reflect the actual onset of psychiatric disorders but rather diagnosis dates. Consequently, determining the temporal sequence of events is not entirely straightforward. Furthermore, patients with assault-related injuries may have a higher likelihood of referral to psychiatric services compared to the general population, which could lead to inflated rates of diagnosed psychiatric disorders in this group. To mitigate the risks for such biases, we initiated the follow-up period up to 2 years after the initial victimisation event in complementary sensitivity tests and found that the results were commensurate with the main findings. This time lag strengthens the argument that the associations are unlikely due to reverse causation bias or solely attributable to earlier referrals to psychiatric services. In addition, we examined premature mortality as outcome, as it is to a lesser extent affected by referral practices and found that it was similarly associated with earlier victimisation experience.

Third, outpatient care data were not available prior to 1998 in Finland and 2001 in Sweden. Nevertheless, our complementary sensitivity analyses, which examined moderation effects by birth cohorts and follow-ups beginning from 2006 onwards, collectively suggested that the potential bias due to reduced coverage for the older cohorts is unlikely to affect our main findings.

Fourth, while the sibling-comparison design is effective in controlling for shared unmeasured familial confounders, it does not address the impact of non-shared confounders [20]. For this reason, some findings could be explained by residual genetic confounders that are not shared between siblings. However, we note that the observed within-family associations remain substantial. We also measure a number a previously suggested nongenetic confounders not shared between siblings, but observed in our data. Adjustments for such factors, such as low family income and having a single mother at birth, also increase the credibility of our findings. It is nevertheless important to note that sibling-comparison estimates may underestimate causal effects in the presence of misclassification bias, shared mediators, and sibling carryover effects [32,20]. Future studies may benefit from triangulating our findings with alternative genetically informative research designs [33].

The generalisability of our findings is supported by 2 findings. First, rates of psychiatric disorders are similar across western European countries [34], and self-reported violent victimisation rates in Finland and Sweden were closely aligned with the global average during the period 2003 to 2004 (2.2% to 3.5% versus 3.1%) when the last international comparison was conducted [35]. Second, the country-specific associations in the present study were not systematically different from one another.

Our findings underline the importance of routinely considering how to prevent and manage victimisation risk in mental health services, and also the role of general hospital and emergency medicine to address mental health and re-victimisation. This will entail assessing risks in a structured and consistent way, which will enable stratification, allocation of additional resources to individuals at elevated risks, and potentially collaborative risk management with patients. Furthermore, research exploring the optimisation of trauma-based interventions could potentially reduce risks of post-victimisation risks of common psychiatric disorders and premature mortality. This includes investigations into tailoring therapies to specific populations and settings and evaluating the long-term effectiveness of existing approaches. Public health strategies can further broaden the scope beyond individual interventions, fostering closer collaboration with social services and criminal justice, and advocating for public policies targeting systemic factors like alcohol and drug misuse, which contribute to wider societal victimisation risks.

In summary, in this large cohort study, we found that individuals who experienced violent victimisation were at least twice as likely as their unaffected siblings to be diagnosed with common psychiatric disorders, to engage in suicidal behaviours, and to die prematurely. These risks remained elevated over 2 years post-victimisation, suggesting that mental health services should review how they assess and manage subsequent risks of adverse outcomes.

## Supporting information

S1 Supporting information Diagnostic validity Additional sensitivity tests. **Checklist**. STROBE statement. **Table A**. ICD diagnostic codes. **Table B**. Measured confounders. **Table C**. Baseline demographic characteristics in Finland. **Table D**. Baseline demographic characteristics in Sweden. **Table E**. Person-time at risk, number of individuals with the outcomes, and incident rates per 1,000 person-years for common psychiatric disorders, suicidal behaviours, and premature mortality, stratified across individuals exposed to violent victimisation in Finland and Sweden. **Table F**. Person-time at risk, number of individuals with the outcomes, and incident rates per 1,000 person-years for common psychiatric disorders, suicidal behaviours, and premature mortality, stratified across exposure to violent victimisation and sex. **Table G**. Interaction terms between violent victimisation and preexisting psychiatric disorders and self-harm on subsequent risk for common psychiatric disorders, suicidal behaviours, and premature mortality in Finland and Sweden. **Figure A**. Country-specific within-family associations between violent victimisation and subsequent common psychiatric disorders, suicidal behaviours, and premature mortality in Finland and Sweden. **Figure B**. Cohort-specific within-family associations between violent victimisation and subsequent common psychiatric disorders, suicidal behaviours, and premature mortality in Finland and Sweden. **Figure C**. Age-specific within-family associations between violent victimisation and subsequent common psychiatric disorders, suicidal behaviours, and premature mortality in Finland and Sweden. **Figure D**. Sex-specific within-family associations between violent victimisation and subsequent common psychiatric disorders, suicidal behaviours, and premature mortality in Finland and Sweden. **Figure E**. Within-family associations between violent victimisation and subsequent common psychiatric disorders, suicidal behaviours, and premature mortality in Finland and Sweden with varying washout periods (e.g., 1 month, 6 months, 12 months, and 24 months). **Figure F**. Within-family associations between violent victimisation and subsequent common psychiatric disorders, suicidal behaviours, and premature mortality in Finland and Sweden and adjusted for additional indicators for parental socioeconomic status (SES). **Figure G**. Within-family associations between violent victimisation and subsequent common psychiatric disorders and suicidal behaviours (at least 2 diagnoses on separate occasions) in Finland and Sweden. **Figure H**. Within-family associations between violent victimisation and subsequent common psychiatric disorders, suicidal behaviours, and premature mortality in Finland and Sweden stratified across the entire period and the most recent period (2006–2020). **Figure I**. Within-family associations between violent victimisation and subsequent common psychiatric disorders, suicidal behaviours, and premature mortality in Finland and Sweden, excluding individuals with preexisting psychiatric disorders. **Figure J**. Population-wide and within-family associations between violent victimisation and subsequent stress-related disorders in Finland and Sweden.
(DOCX)

## Abbreviations

aHR: adjusted hazard ratio
CI: confidence interval
ICD: International Classification of Diseases
SES: socioeconomic status
