# Supplementary material for: Risk of common psychiatric disorders, suicidal behaviours, and premature mortality following violent victimisation: A matched cohort and sibling-comparison study of 127,628 people who experienced violence in Finland and Sweden
Source: PLoS Med. 2024 Oct 18;21(10):e1004410. doi: 10.1371/journal.pmed.1004410 (PMC11488697; doi:10.1371/journal.pmed.1004410)
Supplement: S1 Supporting information — Checklist. STROBE statement. Table A. ICD diagnostic codes. Table B. Measured confounders. Table C. Baseline demographic characteristics in Finland. Table D. Baseline demographic characteristics in Sweden. Table E. Person-time at risk, number of individuals with the outcomes, and incident rates per 1,000 person-years for common psychiatric disorders, suicidal behaviours, and premature mortality, stratified across individuals exposed to violent victimisation in Finland and Sweden. Table F. Person-time at risk, number of individuals with the outcomes, and incident rates per 1,000 person-years for common psychiatric disorders, suicidal behaviours, and premature mortality, stratified across exposure to violent victimisation and sex. Table G. Interaction terms between violent victimisation and preexisting psychiatric disorders and self-harm on subsequent risk for common psychiatric disorders, suicidal behaviours, and premature mortality in Finland and Sweden. Figure A. Country-specific within-family associations between violent victimisation and subsequent common psychiatric disorders, suicidal behaviours, and premature mortality in Finland and Sweden. Figure B. Cohort-specific within-family associations between violent victimisation and subsequent common psychiatric disorders, suicidal behaviours, and premature mortality in Finland and Sweden. Figure C. Age-specific within-family associations between violent victimisation and subsequent common psychiatric disorders, suicidal behaviours, and premature mortality in Finland and Sweden. Figure D. Sex-specific within-family associations between violent victimisation and subsequent common psychiatric disorders, suicidal behaviours, and premature mortality in Finland and Sweden. Figure E. Within-family associations between violent victimisation and subsequent common psychiatric disorders, suicidal behaviours, and premature mortality in Finland and Sweden with varying washout periods (e.g., 1 month, 6 months, 12 months, and 24 [file pmed.1004410.s001.docx]

**S1 Supporting Information**

**Text A.** Diagnostic validity

**Text B.** Additional sensitivity tests

**Checklist A.** STROBE statement

**Table A.** ICD diagnostic codes

**Table B.** Measured confounders

**Table C.** Baseline demographic characteristics in Finland

**Table D.** Baseline demographic characteristics in Sweden

**Table E.** Person-time at risk, number of individuals with the outcomes, and incident rates per 1000 person-years for common psychiatric disorders, suicidal behaviours, and premature mortality, stratified across individuals exposed to violent victimisation in Finland and Sweden

**Table F.** Person-time at risk, number of individuals with the outcomes, and incident rates per 1000 person-years for common psychiatric disorders, suicidal behaviours, and premature mortality, stratified across exposure to violent victimisation and sex

**Table G.** Interaction terms between violent victimisation and pre-existing psychiatric disorders and self-harm on subsequent risk for common psychiatric disorders, suicidal behaviours and premature mortality in Finland and Sweden

**Figure A.** Country-specific within-family associations between violent victimisation and subsequent common psychiatric disorders, suicidal behaviours and premature mortality in Finland and Sweden

**Figure B.** Cohort-specific within-family associations between violent victimisation and subsequent common psychiatric disorders, suicidal behaviours and premature mortality in Finland and Sweden

**Figure C.** Age-specific within-family associations between violent victimisation and subsequent common psychiatric disorders, suicidal behaviours and premature mortality in Finland and Sweden

**Figure D.** Sex-specific within-family associations between violent victimisation and subsequent common psychiatric disorders, suicidal behaviours and premature mortality in Finland and Sweden

**Figure E.** Within-family associations between violent victimisation and subsequent common psychiatric disorders, suicidal behaviours and premature mortality in Finland and Sweden with varying washout periods (e.g., 1 month, 6 months, 12 months, and 24 months)

**Figure F.** Within-family associations between violent victimisation and subsequent common psychiatric disorders, suicidal behaviours and premature mortality in Finland and Sweden and adjusted for additional indicators for parental socioeconomic status (SES)

**Figure G.** Within-family associations between violent victimisation and subsequent common psychiatric disorders and suicidal behaviours (at least two diagnoses on separate occasions) in Finland and Sweden

**Figure H.** Within-family associations between violent victimisation and subsequent common psychiatric disorders, suicidal behaviours and premature mortality in Finland and Sweden stratified across the entire period and the most recent period (2006-2020)

**Figure I.** Within-family associations between violent victimisation and subsequent common psychiatric disorders, suicidal behaviours, and premature mortality in Finland and Sweden, excluding individuals with pre-existing psychiatric disorders

**Figure J.** Population-wide and within-family associations between violent victimisation and subsequent stress-related disorders in Finland and Sweden

**Text A. Diagnostic validity**

Review studies of the inpatient care diagnoses in the Nordic patient registers have typically reported excellent validity, with positive predictive values (PPVs) typically ranging between 75-99% in Finland and 85-95% in Sweden. Although no specific studies have been conducted to evaluate the validity of violent victimisation diagnoses, the validity of related unintentional injury diagnoses is well established in Finland (PPVs: 91%-96%)^1^ and Sweden (PPV=95%).^2^ Violent victimisation diagnoses have nevertheless been widely utilised in epidemiological studies conducted in the Nordic countries.^3–7^

Depression diagnoses exhibit moderate validity when compared to clinical forensic registers in Sweden (κ=0.31, 88% agreement)^8^ and strong validity when compared to researcher assessments in Finland (κ=0.65, PPV=0.81).^9^ Similarly, Swedish studies of specific anxiety-related disorders (PPVs: 86-97%)^10^ and personality disorders (κ=0.85-1.00, 83% agreement)^11^ have revealed excellent validity.

**References**

1. Sund R. Quality of the Finnish Hospital Discharge Register: A systematic review. *Scand J Public Health*. 2012;40(6):505-515.

2. Ludvigsson J, Andersson E, Ekbom A, et al. External review and validation of the Swedish national inpatient register. *BMC Public Health*. 2011;11(1):450.

3. Walter F, Carr M, Mok P, et al. Multiple adverse outcomes following first discharge from inpatient psychiatric care: a national cohort study. *Lancet Psychiatry*. 2019;6(7):582-589.

4. Pitkänen J, Remes H, Aaltonen M, Martikainen P. Experience of maternal and paternal adversities in childhood as determinants of self-harm in adolescence and young adulthood. *J Epidemiol Community Health*. 2019;73(11):1040-1046.

5. Sariaslan A, Arseneault L, Larsson H, Lichtenstein P, Fazel S. Risk of Subjection to Violence and Perpetration of Violence in Persons With Psychiatric Disorders in Sweden. *JAMA Psychiatry*. 2020;77(4):359-367.

6. Sariaslan A, Kääriälä A, Pitkänen J, et al. Long-term Health and Social Outcomes in Children and Adolescents Placed in Out-of-Home Care. *JAMA Pediatr*. 2022;176(1):e214324.

7. Sariaslan A, Larsson H, Hawton K, et al. Physical injuries as triggers for self-harm: a within-individual study of nearly 250 000 injured people with a major psychiatric disorder. *BMJ Ment Health*. 2023;26(1):e300758.

8. Fazel S, Wolf A, Chang Z, Larsson H, Goodwin GM, Lichtenstein P. Depression and violence: a Swedish population study. *Lancet Psychiatry*. 2015;2(3):224-232.

9. Kieseppä T, Partonen T, Kaprio J, Lönnqvist J. Accuracy of register- and record-based bipolar I disorder diagnoses in Finland; a study of twins. *Acta Neuropsychiatr*. 2000;12(3):106-109.

10. Rück C, Larsson KJ, Lind K, et al. Validity and reliability of chronic tic disorder and obsessive-compulsive disorder diagnoses in the Swedish National Patient Register. *BMJ Open*. 2015;5(6):e007520-e007520.

11. Kouppis E, Ekselius L. Validity of the personality disorder diagnosis in the Swedish National Patient Register. *Acta Psychiatr Scand*. 2020;141(5):432-438.

**Text B. Additional sensitivity tests**

We conducted a number of additional complementary sensitivity analyses to examine the extent to which the estimates were stable under different assumptions.

First, we examined moderation effects by birth cohorts (e.g., those born 1973-1982, 1983-1992 and 1993-2004), age at victimisation categories (e.g., 17 years or younger, 18-24 years, 25-34 years, 35 years or older), and sex by stratifying the sibling-comparison models across each category (**Figures B-D**). With the exception of alcohol use disorders, the estimates were generally similar across different birth cohort and age at victimisation categories. However, people who were subjected to violence aged 25 or older had higher rates of alcohol use disorders compared to younger individuals who were subjected to violence (aHRs: 3.1-4.8 vs 2.4-2.5). Moreover, we found evidence in support of sex differences for all outcomes (aHRs_sisters_: 1.8-3.8; aHRs_brothers_: 1.6-2.5), except premature mortality (aHRs: 1.7-1.7).

To mitigate the potential for bias introduced by unobserved factors that could simultaneously influence the likelihood of being exposed to violence and the development of outcomes during shorter timeframes, we shifted the start date of follow-up between a month, six months, a year, and two years after the initial violent victimisation event. The analyses revealed that the estimates were lightly reduced but were not materially altered from the main findings (**Figure E**).

In Sweden, we had additional data on parental socioeconomic status (i.e., educational attainment, unemployment, disability pension and social assistance benefits) measured at offspring birth for those born 1990-2004. Equivalent data was available for the entire Finish cohort. We therefore re-ran the sibling-comparison models and added these variables but did not find any meaningful differences with the main findings (**Figure F**).

We also considered an alternative outcome definition, where we required that the psychiatric diagnoses had been given on two separate occasions to reduce the risks of obtaining false-positive cases. These findings were commensurate with the main findings (**Figure G)**.

The outpatient care data in the national patient registers started in 1998 in Finland and 2001 in Sweden but it took a couple of years before their coverage was complete. We therefore tested whether restricting our analyses to victimisation events that occurred after 2005 would affect our findings, and we found no meaningful impact (**Figure H**).

We conducted three additional sensitivity analyses post-hoc as part of the review process. First, we examined the extent to which the four binary and one three-level categorical indicators of socioeconomic status measured at birth (i.e., parental low income, unemployment, disability pension, social assistance benefits, and educational attainment) modified associations between violent victimisation and examined outcomes. We found that of the 84 interaction terms estimated, only 3 (3.6%) reached statistical significance (p<0.05) following Bonferroni correction for multiple hypothesis testing. However, none of the three significant interaction terms were independently replicated across both countries.”

Second, we re-ran the main analyses, this time excluding individuals with pre-existing psychiatric disorders instead of adjusting for them. Both approaches yielded nearly identical results (**Figure I**). Third, we examined the following four stress-related disorders as outcome: any stress-related disorder, acute stress response, post-traumatic stress disorder, and adjustment disorders and other stress reactions. As these diagnoses have been recorded from 1996 in Finland and 1987 in Sweden, our analysis was limited to violent victimisation events occurring from these dates. Consistent with the main findings, we found that individuals who had been subjected to violent victimisation were between two to three times as likely as their unaffected siblings to develop any stress-related disorders (aHR: 1.9-3.2; **Figure J**).

**S1 Checklist. STROBE statement**

|  | Item No | Recommendation | Relevant portion of the manuscript |
| --- | --- | --- | --- |
| **Title and abstract** | 1 | (*a*) Indicate the study’s design with a commonly used term in the title or the abstract | Title; Methods section of the Abstract |
|  |  | (*b*) Provide in the abstract an informative and balanced summary of what was done and what was found | Abstract |
| Introduction | | |  |
| Background/rationale | 2 | Explain the scientific background and rationale for the investigation being reported | Introduction, paragraphs 1-4 |
| Objectives | 3 | State specific objectives, including any prespecified hypotheses | Introduction, paragraph 5 |
| Methods | | |  |
| Study design | 4 | Present key elements of study design early in the paper | Introduction, paragraph 5 |
| Setting | 5 | Describe the setting, locations, and relevant dates, including periods of recruitment, exposure, follow-up, and data collection | Methods, paragraphs 1-3 |
| Participants | 6 | (*a*) Give the eligibility criteria, and the sources and methods of selection of participants. Describe methods of follow-up | Methods, paragraphs 4,9 |
|  |  | (*b*) For matched studies, give matching criteria and number of exposed and unexposed | Methods, paragraphs 7,8 |
| Variables | 7 | Clearly define all outcomes, exposures, predictors, potential confounders, and effect modifiers. Give diagnostic criteria, if applicable | Methods, paragraphs 5,6; Tables A-B in S1 Supporting Information |
| Data sources/ measurement | 8* | For each variable of interest, give sources of data and details of methods of assessment (measurement). Describe comparability of assessment methods if there is more than one group | Table B in S1 Supporting Information |
| Bias | 9 | Describe any efforts to address potential sources of bias | Text B in S1 Supporting Information |
| Study size | 10 | Explain how the study size was arrived at | Methods, paragraph 3 |
| Quantitative variables | 11 | Explain how quantitative variables were handled in the analyses. If applicable, describe which groupings were chosen and why | Table B in S1 Supporting Information |
| Statistical methods | 12 | (*a*) Describe all statistical methods, including those used to control for confounding | Methods, paragraphs 9,10 |
|  |  | (*b*) Describe any methods used to examine subgroups and interactions | Text B in S1 Supporting Information |
|  |  | (*c*) Explain how missing data were addressed | Methods, paragraph 3 |
|  |  | (*d*) If applicable, explain how loss to follow-up was addressed | Methods, paragraphs 8,9 |
|  |  | (*e*) Describe any sensitivity analyses | Methods, paragraphs 11,13; Text B in S1 Supporting Information |
| Results | | |  |
| Participants | 13* | (a) Report numbers of individuals at each stage of study—eg numbers potentially eligible, examined for eligibility, confirmed eligible, included in the study, completing follow-up, and analysed | Methods, paragraph 3; Results, paragraph 1 |
|  |  | (b) Give reasons for non-participation at each stage | Methods, paragraph 3; |
|  |  | (c) Consider use of a flow diagram | - |
| Descriptive data | 14* | (a) Give characteristics of study participants (eg demographic, clinical, social) and information on exposures and potential confounders | Results, paragraph 1 |
|  |  | (b) Indicate number of participants with missing data for each variable of interest | Methods, paragraph 3 |
|  |  | (c) Summarise follow-up time (eg, average and total amount) | Results, paragraph 2; Table 2 |
| Outcome data | 15* | Report numbers of outcome events or summary measures over time | Results, paragraph 2; Table 2 |
| Main results | 16 | (*a*) Give unadjusted estimates and, if applicable, confounder-adjusted estimates and their precision (eg, 95% confidence interval). Make clear which confounders were adjusted for and why they were included | Results, paragraph 2 |
|  |  | (*b*) Report category boundaries when continuous variables were categorized | N/A |
|  |  | (*c*) If relevant, consider translating estimates of relative risk into absolute risk for a meaningful time period | We present incidence rates in Table 2 |
| Other analyses | 17 | Report other analyses done—eg analyses of subgroups and interactions, and sensitivity analyses | Results, paragraphs 3-5 |
| Discussion | | |  |
| Key results | 18 | Summarise key results with reference to study objectives | Discussion, paragraphs 1-6 |
| Limitations | 19 | Discuss limitations of the study, taking into account sources of potential bias or imprecision. Discuss both direction and magnitude of any potential bias | Discussion, paragraphs 9-12 |
| Interpretation | 20 | Give a cautious overall interpretation of results considering objectives, limitations, multiplicity of analyses, results from similar studies, and other relevant evidence | Discussion, paragraphs 14-15 |
| Generalisability | 21 | Discuss the generalisability (external validity) of the study results | Discussion, paragraph 13 |
| Other information | | |  |
| Funding | 22 | Give the source of funding and the role of the funders for the present study and, if applicable, for the original study on which the present article is based | Acknowledgment |

**Table A. ICD diagnostic codes**

|  | **ICD-8** | **ICD-9** | **ICD-10** |
| --- | --- | --- | --- |
| **Violent victimisation** | E960-E969 | E960-E969 | X85-X99, Y00-Y09 |
|  |  |  |  |
| **Any psychiatric disorder** | 290-315 | 290-319 | F00-F99 |
| **Depression** |  |  |  |
| Finland | 296.2, 300.4 | 296.1 [excl. 296.1E],  296.8A, 300.4A | F32-F39  [excl. F32.3 and F33.3] |
| Sweden | 296.2, 300.4 | 296B, 300E, 311 | F32-F39  [excl. F32.3 and F33.3] |
| **Anxiety** |  |  |  |
| Finland | 300 except 300.4 | 300 except 300.4A | F40-F42, F44-F45, F48 |
| Sweden | 300 except 300.4 | 300 except 300E | F40-F42, F44-F45, F48 |
| **Personality disorder** | 301 | 301 | F60-F69 |
| **Alcohol use disorder** |  |  |  |
| Finland | 291, 303 | 291, 303, 305.0 | F10 [excl. F10.5] |
| Sweden | 291, 303 | 291, 303, 305A | F10 [excl. F10.5] |
| **Drug use disorder** |  |  |  |
| Finland | 304 | 292, 304, 305.2-305.9 | F11-F12, F14-F16, F19  [excl. F1*.5] |
| Sweden | 304 | 292, 304, 305X | F11-F12, F14-F16, F19  [excl. F1*.5] |
| **Suicidal behaviour** |  |  |  |
| Finland | - | E950A-E959X,  E970A-E979A | X60-X84,  Y10-Y34 |
| Sweden | E950-E959,  E980-E989 | E950-E959,  E980-E989 | X60-X84,  Y10-Y34 |
| **Any stress-related disorder** |  |  |  |
| Finland | - | - | F43 |
| Sweden | - | 308, 309 | F43 |
| **Acute stress reaction** |  |  |  |
| Finland | - | - | F43.0 |
| Sweden | - | 308, 309A | F43.0 |
| **Post-traumatic stress disorder** |  |  |  |
| Finland | - | - | F43.1 |
| Sweden | - | 309B | F43.1 |
| **Adjustment disorder and other stress reactions** |  |  |  |
| Finland | - | - | F43.2, F43.8, F43.9 |
| Sweden | - | 309X | F43.2, F43.8, F43.9 |

**Table B. Measured confounders**

| **Measured confounder** | **Definition** |
| --- | --- |
| Sex | A binary indicator (0=Male; 1=Female) |
| Birth year | Categorical measure with 18 levels (1987-2004) in Finland and 32 levels (1973-2004) in Sweden. |
| Birth order | Categorical measure with 4 levels based on the order of birth within each mother: 1=First-born, 2= second-born, 3=third-born, 4=fourth-born or higher. |
| Immigrant background | A binary indicator (0=Both parents were born in Finland or Sweden; 1=At least one parent was born abroad). |
| Single mother at birth | A binary indicator of whether the individual resided with a single mother at the end of their birth year. |
| Low family income at birth | A binary indicator of whether the sum of the inflation-adjusted income of both biological parents measured at the birth of the offspring fell below the bottom decile of the sample. If data were missing, we chose the first available measurement.  In Finland, we were able to obtain gross income data for the entire period and disposable income data from 1995 onwards, while in Sweden, we only had disposable income data. To create these binary measures, we used gross income data in Finland and disposable income data in Sweden. As a complementary sensitivity test, we investigated the correlation between gross income and disposable income measures in Finland for those born between 1995 and 2004 and discovered that it was nearly identical (r=0.97). |
| Parental psychiatric disorders at birth | A binary indicator of whether any of the parents had been diagnosed with any psychiatric disorder prior to offspring birth. |
| Parental violent crime | A binary indicator of whether any of the parents had been convicted of a violent crime prior to offspring birth. |
| Pre-existing psychiatric disorders and self-harm | We used the same definitions as the outcome measures in the study, but measured these confounders before the victimisation event (or the equivalent time period for the unexposed individuals). We also adjusted for a binary measure of any other psychiatric disorders that had been diagnosed prior to the violent victimisation event or the equivalent time period for unaffected individuals. |
|  |  |
| **Adjustments in complementary sensitivity analyses:** |  |
| Parental educational attainment at birth | The highest level of education achieved by either parent in three categories: 1=primary level (International Standard Classification of Education [ISCED] 2011 levels: 0-2), 2=secondary level (ISCED 2011 levels: 3-5) or 3=tertiary level (ISCED 2011 levels: 6-8). Measured at the end of the year in which the offspring was born. |
| Parental unemployment at birth | A binary indicator of whether any of the parents had been unemployed at least one day during the year in which the offspring was born. |
| Parental disability pension at birth | A binary indicator of whether any of the parents had been unemployed at least one day during the year in which the offspring was born. |
| Parental social assistance benefits at birth | A binary indicator of whether any of the parents had been recipients of social assistance benefits during the year in which the offspring was born. |

**Table C. Baseline demographic characteristics in Finland**

|  | **General population controls** | **Individuals exposed to violent victimisation** | **Unaffected**  **sibling controls** | **Individuals exposed to violent victimisation with unaffected siblings** |
| --- | --- | --- | --- | --- |
| **Total, n** | 194,240 | 19,424 | 20,425 | 12,711 |
| **Median age at victimisation (IQR) years** | - | 21 (18-24) | - | 21 (18-24) |
| **Sex, n (%)** |  |  |  |  |
| Female | 55,520 (28.6%) | 5552 (28.6%) | 10,134 (49.6%) | 3563 (28.0%) |
| Male | 138,720 (71.4%) | 13,872 (71.4%) | 10,291 (50.4%) | 9148 (72.0%) |
| **Birth cohorts, n (%)** |  |  |  |  |
| 1973-1974 | - | - | - | - |
| 1975-1979 | - | - | - | - |
| 1980-1984 | - | - | - | - |
| 1985-1989 | 46,420 (23.9%) | 4642 (23.9%) | 3228 (15.8%) | 2378 (18.7%) |
| 1990-1994 | 71,670 (36.9%) | 7167 (36.9%) | 7540 (36.9%) | 5072 (39.9%) |
| 1995-1999 | 52,120 (26.8%) | 5212 (26.8%) | 6150 (30.1%) | 3849 (30.3%) |
| 2000-2004 | 24,030 (12.4%) | 2403 (12.4%) | 3507 (17.2%) | 1412 (11.1%) |
| **Birth order, n (%)** |  |  |  |  |
| 1st | 76,558 (39.4%) | 7412 (38.2%) | 5133 (25.1%) | 4323 (34.0%) |
| 2nd | 67,069 (34.5%) | 6701 (34.5%) | 6384 (31.3%) | 4792 (37.7%) |
| 3rd | 32,601 (16.8%) | 3396 (17.5%) | 4576 (22.4%) | 2222 (17.5%) |
| 4th or higher | 18,012 (9.3%) | 1915 (9.9%) | 4332 (21.2%) | 1374 (10.8%) |
| **Immigration background, n (%)** | 9388 (4.8%) | 1382 (7.1%) | 1423 (7.0%) | 831 (6.5%) |
| **Mother single at offspring birth, n (%)** | 52,320 (26.9%) | 7369 (37.9%) | 5610 (27.5%) | 4108 (32.3%) |
| **Family income in the bottom decile at offspring birth, n (%)** | 18,091 (9.3%) | 3293 (17.0%) | 2911 (14.3%) | 1886 (14.8%) |
| **Parental history of psychiatric disorders, n (%)** | 12,901 (6.6%) | 2515 (12.9%) | 2323 (11.4%) | 1399 (11.0%) |
| **Parental history of violent criminality, n (%)** | 13,948 (7.2%) | 3396 (17.5%) | 3090 (15.1%) | 2004 (15.8%) |
| **Pre-existing psychiatric disorders and self-harm, n (%)** |  |  |  |  |
| Depression | 10,652 (5.5%) | 3000 (15.4%) | 1772 (8.7%) | 1852 (14.6%) |
| Anxiety | 9232 (4.8%) | 2587 (13.3%) | 1412 (6.9%) | 1588 (12.5%) |
| Personality disorder | 1678 (0.9%) | 709 (3.7%) | 265 (1.3%) | 423 (3.3%) |
| Alcohol use disorder | 3215 (1.7%) | 1935 (10.0%) | 687 (3.4%) | 1214 (9.6%) |
| Drug use disorder | 1292 (0.7%) | 1324 (6.8%) | 309 (1.5%) | 772 (6.1%) |
| Other psychiatric disorders | 19,636 (10.1%) | 2895 (14.9%) | 2461 (12.0%) | 1812 (14.3%) |
| Self-harm | 1919 (1.0%) | 1353 (7.0%) | 433 (2.1%) | 817 (6.4%) |

*Notes: IQR refers to interquartile range.*

**Table D. Baseline demographic characteristics in Sweden**

|  | **General population controls** | **Individuals exposed to violent victimisation** | **Unaffected**  **sibling controls** | **Individuals exposed to violent victimisation with unaffected siblings** |
| --- | --- | --- | --- | --- |
| **Total, n** | 1,081,975 | 108,204 | 111,983 | 73,114 |
| **Median age at victimisation (IQR) years** | - | 21 (18-26) | - | 21 (18-25) |
| **Sex, n (%)** |  |  |  |  |
| Female | 340,470 (31.5%) | 34,047 (31.5%) | 55,914 (49.9%) | 22,151 (30.3%) |
| Male | 741,505 (68.5%) | 74,157 (68.5%) | 56,069 (50.1%) | 50,963 (69.7%) |
| **Birth cohorts, n (%)** |  |  |  |  |
| 1973-1974 | 51,940 (4.8%) | 5194 (4.8%) | 3,446 (3.1%) | 1,990 (2.7%) |
| 1975-1979 | 134,290 (12.4%) | 13,429 (12.4%) | 13,021 (11.6%) | 7,820 (10.7%) |
| 1980-1984 | 179,270 (16.6%) | 17,927 (16.6%) | 19,316 (17.2%) | 12,495 (17.1%) |
| 1985-1989 | 260,642 (24.1%) | 26,070 (24.1%) | 26,720 (23.9%) | 19,152 (26.2%) |
| 1990-1994 | 255,583 (23.6%) | 25,559 (23.6%) | 26,979 (24.1%) | 18,628 (25.5%) |
| 1995-1999 | 132,200 (12.2%) | 13,220 (12.2%) | 14,590 (13.0%) | 9,221 (12.6%) |
| 2000-2004 | 68,050 (6.3%) | 6805 (6.3%) | 7,911 (7.1%) | 3,808 (5.2%) |
| **Birth order, n (%)** |  |  |  |  |
| 1st | 697,199 (64.4%) | 67,760 (62.6%) | 60,436 (54%) | 42,890 (58.7%) |
| 2nd | 299,091 (27.6%) | 29,918 (27.6%) | 34,555 (30.9%) | 22,089 (30.2%) |
| 3rd | 68,992 (6.4%) | 8141 (7.5%) | 12,100 (10.8%) | 6234 (8.5%) |
| 4th or higher | 16,693 (1.5%) | 2385 (2.2%) | 4892 (4.4%) | 1901 (2.6%) |
| **Immigration background, n (%)** | 187,909 (17.4%) | 25,113 (23.2%) | 25,235 (22.5%) | 15,740 (21.5%) |
| **Mother single at offspring birth, n (%)** | 468,243 (43.3%) | 55,820 (51.6%) | 47,800 (42.7%) | 33,662 (46.0%) |
| **Family income in the bottom decile at offspring birth, n (%)** | 100,968 (9.3%) | 13,725 (12.7%) | 10,254 (9.2%) | 7446 (10.2%) |
| **Parental history of psychiatric disorders, n (%)** | 41,585 (3.8%) | 8866 (8.2%) | 7271 (6.5%) | 4649 (6.4%) |
| **Parental history of violent criminality, n (%)** | 53,317 (4.9%) | 13,080 (12.1%) | 11,554 (10.3%) | 7,435 (10.2%) |
| **Pre-existing psychiatric disorders and self-harm, n (%)** |  |  |  |  |
| Depression | 31,711 (2.9%) | 10,327 (9.5%) | 5061 (4.5%) | 6448 (8.8%) |
| Anxiety | 37,146 (3.4%) | 12,092 (11.2%) | 5701 (5.1%) | 7421 (10.1%) |
| Personality disorder | 6187 (0.6%) | 3710 (3.4%) | 1217 (1.1%) | 2159 (3.0%) |
| Alcohol use disorder | 20,641 (1.9%) | 10,190 (9.4%) | 3659 (3.3%) | 6244 (8.5%) |
| Drug use disorder | 9307 (0.9%) | 8173 (7.6%) | 1959 (1.7%) | 4730 (6.5%) |
| Other psychiatric disorders | 49,020 (4.5%) | 9033 (8.3%) | 6269 (5.6%) | 5682 (7.8%) |
| Self-harm | 21,476 (2.0%) | 9130 (8.4%) | 3662 (3.3%) | 5565 (7.6%) |

*Notes: IQR refers to interquartile range.*

**Table E. Person-time at risk, number of individuals with the outcomes, and incident rates per 1000 person-years for common psychiatric disorders, suicidal behaviours, and premature mortality, stratified across individuals exposed to violent victimisation in Finland and Sweden**

| **Outcome/Country** | **Exposure to violent victimisation** | **Number of individuals, n** | **Person-years at risk, years (mean)** | **Number of individuals with the outcome, n** | **Incidence rate per 1000 person-years [95% CI]** |
| --- | --- | --- | --- | --- | --- |
| ***Depression*** |  |  |  |  |  |
| Finland | Unexposed | 194,240 | 1,103,109 (5.7) | 10,680 | 9.7 [9.5; 9.9] |
|  | Exposed | 19,424 | 102,071 (5.3) | 2733 | 26.8 [25.8; 27.8] |
|  |  |  |  |  |  |
| Sweden | Unexposed | 1,081,975 | 10,808,307 (10.0) | 61,964 | 5.7 [5.7; 5.8] |
|  | Exposed | 108,204 | 1,000,632 (9.2) | 16,392 | 16.4 [16.1; 16.6] |
|  |  |  |  |  |  |
| ***Anxiety*** |  |  |  |  |  |
| Finland | Unexposed | 194,240 | 1,109,283 (5.7) | 9949 | 9.0 [8.8; 9.1] |
|  | Exposed | 19,424 | 103,242 (5.3) | 2807 | 27.2 [26.2; 28.2] |
|  |  |  |  |  |  |
| Sweden | Unexposed | 1,081,975 | 10,738,529 (9.9) | 76,890 | 7.2 [7.1; 7.2] |
|  | Exposed | 108,204 | 975,572 (9.0) | 21,520 | 22.1 [21.8; 22.4] |
|  |  |  |  |  |  |
| ***Personality disorder*** |  |  |  |  |  |
| Finland | Unexposed | 194,240 | 1,137,067 (5.9) | 2464 | 2.2 [2.1; 2.3] |
|  | Exposed | 19,424 | 110,510 (5.7) | 925 | 8.4 [7.8; 8.9] |
|  |  |  |  |  |  |
| Sweden | Unexposed | 1,081,975 | 11,104,226 (10.3) | 14,215 | 1.3 [1.3; 1.3] |
|  | Exposed | 108,204 | 1,062,487 (9.8) | 6911 | 6.5 [6.4; 6.7] |
|  |  |  |  |  |  |
| ***Alcohol use disorder*** |  |  |  |  |  |
| Finland | Unexposed | 194,240 | 1,133,314 (5.8) | 3215 | 2.8 [2.7; 2.9] |
|  | Exposed | 19,424 | 104,690 (5.4) | 2141 | 20.5 [19.6; 21.3] |
|  |  |  |  |  |  |
| Sweden | Unexposed | 1,081,975 | 11,025,675 (10.2) | 24,797 | 2.2 [2.2; 2.3] |
|  | Exposed | 108,204 | 1,009,237 (9.3) | 13,844 | 13.7 [13.5; 13.9] |
| ***Drug use disorder*** |  |  |  |  |  |
| Finland | Unexposed | 194,240 | 1,139,172 (5.9) | 1907 | 1.7 [1.6; 1.8] |
|  | Exposed | 19,424 | 107,326 (5.5) | 1944 | 18.1 [17.3; 18.9] |
|  |  |  |  |  |  |
| Sweden | Unexposed | 1,081,975 | 11,074,953 (10.2) | 20,065 | 1.8 [1.8; 1.8] |
|  | Exposed | 108,204 | 1,015,639 (9.4) | 14,814 | 14.6 [14.4; 14.8] |
|  |  |  |  |  |  |
| ***Suicidal behaviours*** |  |  |  |  |  |
| Finland | Unexposed | 194,240 | 1,139,228 (5.9) | 1882 | 1.7 [1.6; 1.7] |
|  | Exposed | 19,424 | 107,769 (5.5) | 1552 | 14.4 [13.7; 15.1] |
|  |  |  |  |  |  |
| Sweden | Unexposed | 1,081,975 | 11,041,155 (10.2) | 22,620 | 2.0 [2.0; 2.1] |
|  | Exposed | 108,204 | 1,030,726 (9.5) | 11,409 | 11.1 [10.9; 11.3] |
|  |  |  |  |  |  |
| ***Premature mortality*** |  |  |  |  |  |
| Finland | Unexposed | 194,240 | 1,146,524 (5.9) | 699 | 0.6 [0.6; 0.7] |
|  | Exposed | 19,424 | 114,423 (5.9) | 333 | 2.9 [2.6; 3.2] |
|  |  |  |  |  |  |
| Sweden | Unexposed | 1,081,975 | 11,194,709 (10.3) | 6390 | 0.6 [0.6; 0.6] |
|  | Exposed | 108,204 | 1,109,314 (10.3) | 2473 | 2.2 [2.1; 2.3] |

*Notes: CI refers to confidence intervals.*

**Table F. Person-time at risk, number of individuals with the outcomes, and incident rates per 1000 person-years for common psychiatric disorders, suicidal behaviours, and premature mortality, stratified across exposure to violent victimisation and sex**

| **Outcome/Country** | **Exposure to violent victimisation** | **Number of individuals, n** | **Person-years at risk, years (mean)** | **Number of individuals with the outcome, n** | **Incidence rate per 1000 person-years [95% CI]** |
| --- | --- | --- | --- | --- | --- |
| ***Depression*** |  |  |  |  |  |
| Women | Unexposed | 395,990 | 3,258,234 (8.2) | 30,162 | 9.3 [9.2; 9.4] |
|  | Exposed | 39,599 | 283,270 (7.2) | 9498 | 33.5 [32.9; 34.2] |
|  |  |  |  |  |  |
| Men | Unexposed | 880,225 | 8,653,182 (9.8) | 42,482 | 4.9 [4.9; 5.0] |
|  | Exposed | 88,029 | 819,433 (9.3) | 9627 | 11.7 [11.5; 12.0] |
|  |  |  |  |  |  |
| ***Anxiety*** |  |  |  |  |  |
| Women | Unexposed | 395,990 | 3,229,940 (8.2) | 37,461 | 11.6 [11.5; 11.7] |
|  | Exposed | 39,599 | 271,339 (6.9) | 12,248 | 45.1 [44.3; 45.9] |
|  |  |  |  |  |  |
| Men | Unexposed | 880,225 | 8,617,872 (9.8) | 49,378 | 5.7 [5.7; 5.8] |
|  | Exposed | 88,029 | 807,475 (9.2) | 12,079 | 15.0 [14.7; 15.2] |
|  |  |  |  |  |  |
| ***Personality disorder*** |  |  |  |  |  |
| Women | Unexposed | 395,990 | 3,389,833 (8.6) | 7568 | 2.2 [2.2; 2.3] |
|  | Exposed | 39,599 | 313,408 (7.9) | 4620 | 14.7 [14.3; 15.2] |
|  |  |  |  |  |  |
| Men | Unexposed | 880,225 | 8,851,460 (10.1) | 9111 | 1.0 [1.0; 1.1] |
|  | Exposed | 88,029 | 859,589 (9.8) | 3216 | 3.7 [3.6; 3.9] |
|  |  |  |  |  |  |
| ***Alcohol use disorder*** |  |  |  |  |  |
| Women | Unexposed | 395,990 | 3,386,434 (8.6) | 7102 | 2.1 [2.0; 2.1] |
|  | Exposed | 39,599 | 306,399 (7.7) | 5327 | 17.4 [16.9; 17.9] |
|  |  |  |  |  |  |
| Men | Unexposed | 880,225 | 8,772,555 (10.0) | 20,910 | 2.4 [2.4; 2.4] |
|  | Exposed | 88,029 | 807,528 (9.2) | 10,658 | 13.2 [12.9; 13.5] |
| ***Drug use disorder*** |  |  |  |  |  |
| Women | Unexposed | 395,990 | 3,410,369 (8.6) | 4133 | 1.2 [1.2; 1.2] |
|  | Exposed | 39,599 | 312,674 (7.9) | 5134 | 16.4 [16.0; 16.9] |
|  |  |  |  |  |  |
| Men | Unexposed | 880,225 | 8,803,756 (10.0) | 17,839 | 2.0 [2.0; 2.1] |
|  | Exposed | 88,029 | 810,292 (9.2) | 11,624 | 14.3 [14.1; 14.6] |
|  |  |  |  |  |  |
| ***Suicidal behaviours*** |  |  |  |  |  |
| Women | Unexposed | 395,990 | 3,383,542 (8.5) | 7177 | 2.1 [2.1; 2.2] |
|  | Exposed | 39,599 | 305,885 (7.7) | 5347 | 17.5 [17.0; 18.0] |
|  |  |  |  |  |  |
| Men | Unexposed | 880,225 | 8,796,840 (10.0) | 17,325 | 2.0 [1.9; 2.0] |
|  | Exposed | 88,029 | 832,609 (9.5) | 7614 | 9.1 [8.9; 9.4] |
|  |  |  |  |  |  |
| ***Premature mortality*** |  |  |  |  |  |
| Women | Unexposed | 395,990 | 3,433,000 (8.7) | 1079 | 0.3 [0.3; 0.3] |
|  | Exposed | 39,599 | 342,885 (8.7) | 588 | 1.7 [1.6; 1.9] |
|  |  |  |  |  |  |
| Men | Unexposed | 880,225 | 8,908,233 (10.1) | 6010 | 0.7 [0.7; 0.7] |
|  | Exposed | 88,029 | 880,852 (10.0) | 2218 | 2.5 [2.4; 2.6] |

*Notes: CI refers to confidence intervals.*

**Table G. Interaction terms between violent victimisation and pre-existing psychiatric disorders and self-harm on subsequent risk for common psychiatric disorders, suicidal behaviours and premature mortality in Finland and Sweden**

|  | **Finland** | | **Sweden** | | **Pooled** | |
| --- | --- | --- | --- | --- | --- | --- |
|  | **aHR [95% CI]** | **P** | **aHR [95% CI]** | **P** | **aHR [95% CI]** | **P** |
| Depression | 0.9 [0.8; 1.0] | 0.080 | 0.9 [0.8; 0.9] | <0.001 | 0.9 [0.8; 1.0] | 0.002 |
| Anxiety | 0.9 [0.8; 1.1] | 0.205 | 1.0 [0.9; 1.1] | 0.974 | 1.0 [0.9; 1.1] | 0.738 |
| Personality disorder | 1.4 [1.0; 1.9] | 0.048 | 0.9 [0.8; 1.0] | 0.097 | 1.0 [0.8; 1.1] | 0.476 |
| Alcohol use disorder | 1.2 [1.0; 1.6] | 0.056 | 1.4 [1.2; 1.5] | <0.001 | 1.3 [1.2; 1.5] | <0.001 |
| Drug use disorder | 1.1 [0.8; 1.5] | 0.484 | 1.2 [1.1; 1.4] | <0.001 | 1.2 [1.1; 1.4] | <0.001 |
| Suicidal behaviour | 0.9 [0.7; 1.2] | 0.651 | 1.3 [1.2; 1.4] | <0.001 | 1.2 [1.1; 1.4] | <0.001 |
| Premature mortality | 1.2 [0.7; 2.2] | 0.490 | 1.3 [1.0; 1.6] | 0.024 | 1.3 [1.0; 1.6] | 0.059 |

*Notes: aHR refers to adjusted hazard ratios and CI to confidence intervals.*

**Figure A. Country-specific within-family associations between violent victimisation and subsequent common psychiatric disorders, suicidal behaviours and premature mortality in Finland and Sweden**

**
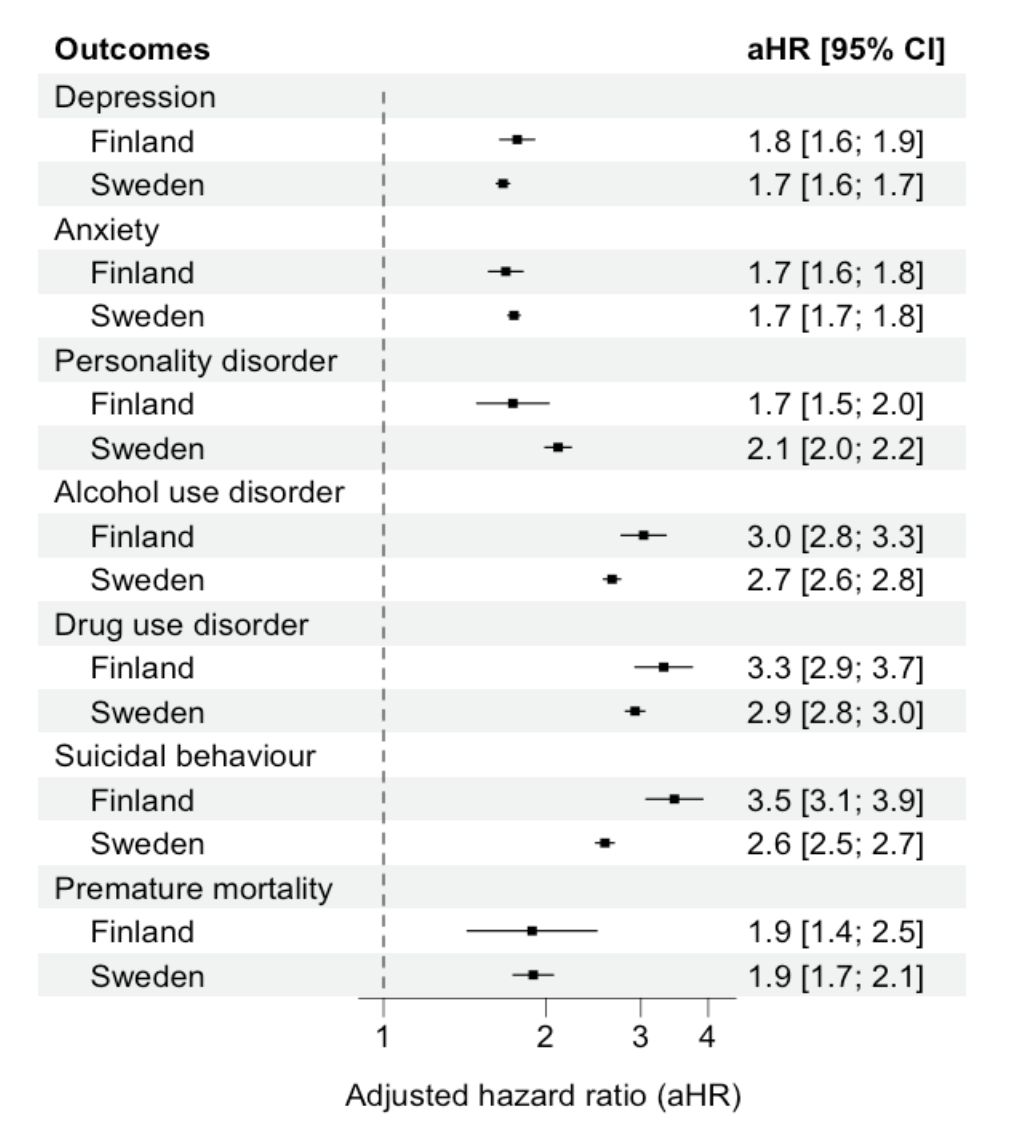
**

*Notes: The estimates refer to within-family associations comparing biological full-siblings differentially exposed to violent victimisation and is adjusted for all time-invariant unmeasured familial confounders shared between the siblings as well as the following measured confounders that vary within families: sex, birth year, birth order, and parental characteristics at birth (low family income, single mother, psychiatric history, and violent crime history) as well as any pre-existing psychiatric disorders and self-harm events. CI refers to confidence intervals.*

**Figure B. Cohort-specific within-family associations between violent victimisation and subsequent common psychiatric disorders, suicidal behaviours, and premature mortality in Finland and Sweden**

**
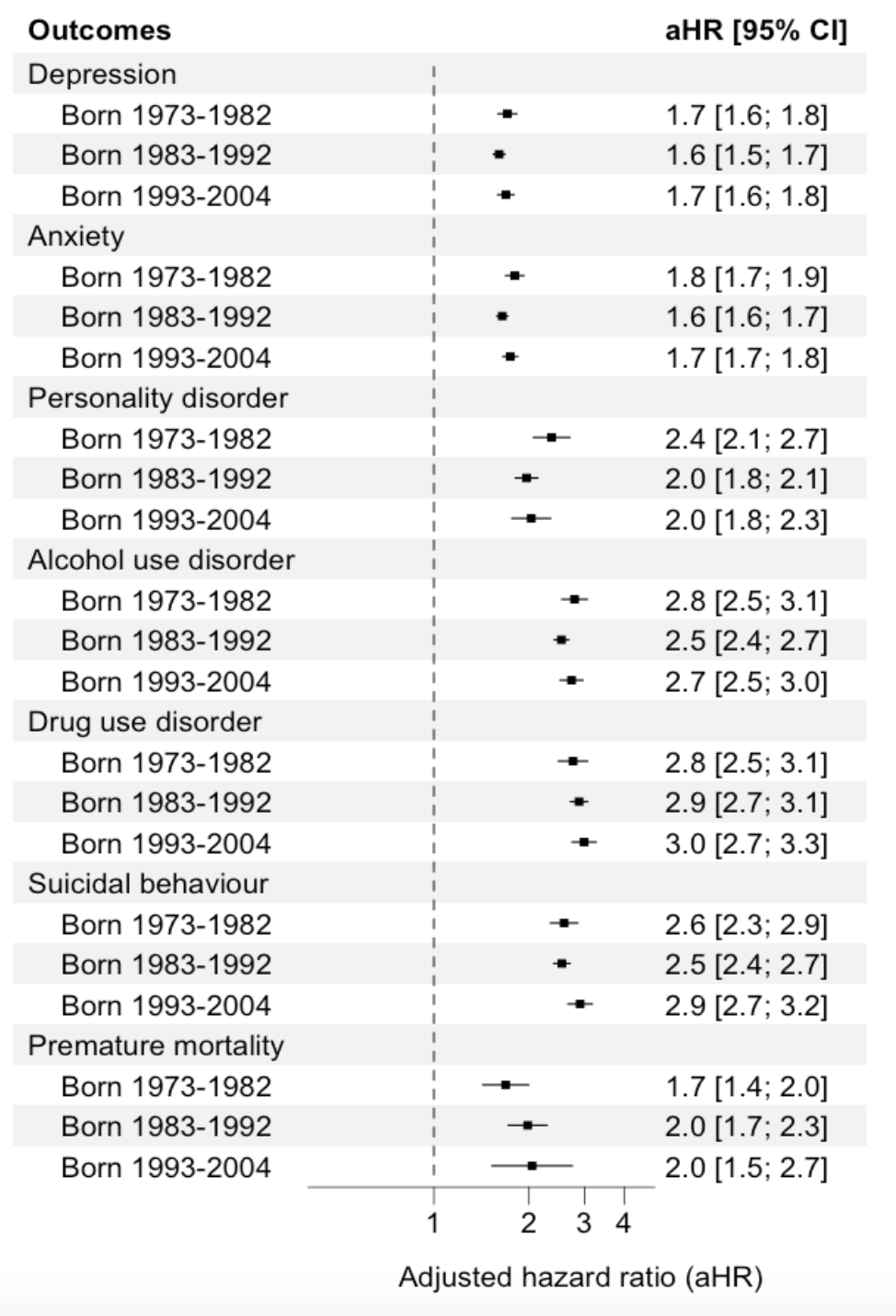
**

*Notes: The estimates refer to within-family associations comparing biological full-siblings differentially exposed to violent victimisation and is adjusted for all time-invariant unmeasured familial confounders shared between the siblings as well as the following measured confounders that vary within families: sex, birth year, birth order, and parental characteristics at birth (low family income, single mother, psychiatric history, and violent crime history) as well as any pre-existing psychiatric disorders and self-harm events. CI refers to confidence intervals.*

**Figure C. Age-specific within-family associations between violent victimisation and subsequent common psychiatric disorders, suicidal behaviours and premature mortality in Finland and Sweden**

*
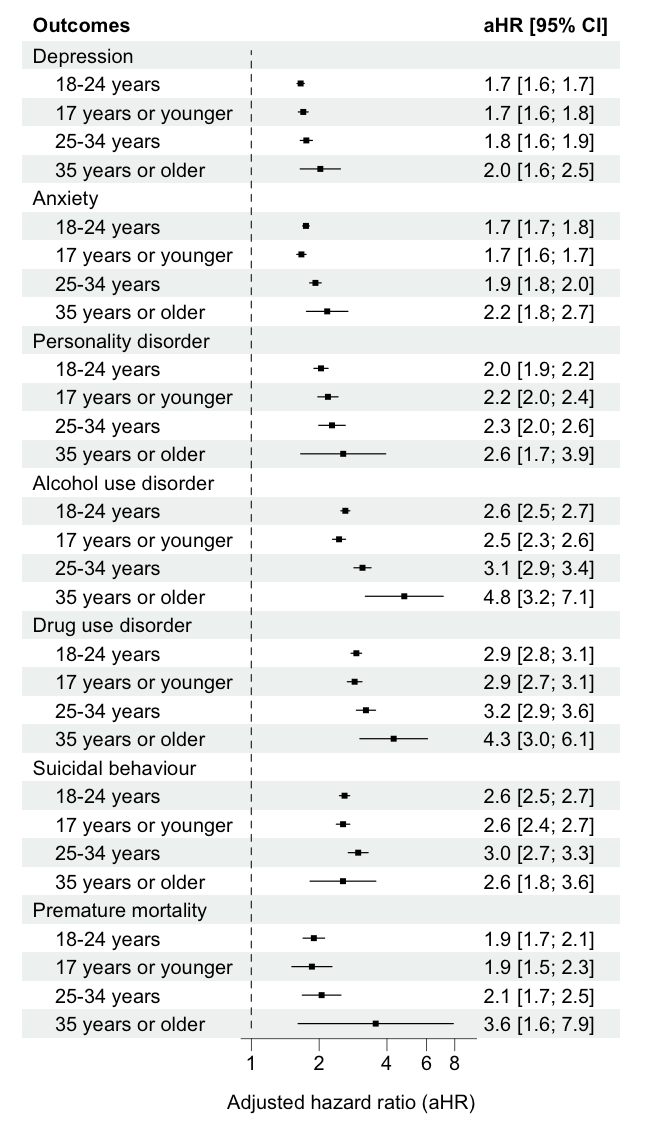
*

*Notes: The estimates refer to within-family associations comparing biological full-siblings differentially exposed to violent victimisation and is adjusted for all time-invariant unmeasured familial confounders shared between the siblings as well as the following measured confounders that vary within families: sex, birth year, birth order, and parental characteristics at birth (low family income, single mother, psychiatric history, and violent crime history) as well as any pre-existing psychiatric disorders and self-harm events. To facilitate model convergence, we used a continuous measure of birth year in the Finnish analyses. CI refers to confidence intervals.*

**Figure D. Sex-specific within-family associations between violent victimisation and subsequent common psychiatric disorders, suicidal behaviours, and premature mortality in Finland and Sweden**

**
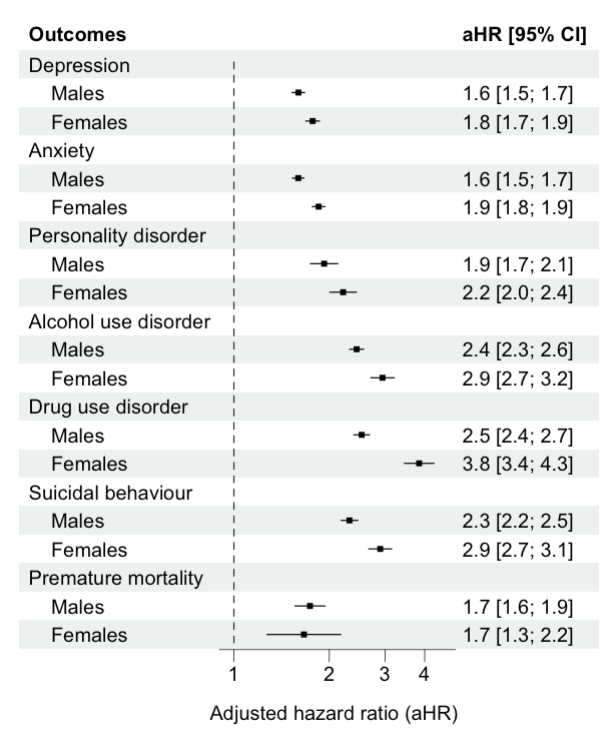
**

*Notes: The estimates refer to within-family associations comparing biological full-siblings differentially exposed to violent victimisation and is adjusted for all time-invariant unmeasured familial confounders shared between the siblings as well as the following measured confounders that vary within families: birth year, birth order, and parental characteristics at birth (low family income, single mother, psychiatric history, and violent crime history) as well as any pre-existing psychiatric disorders and self-harm events. CI refers to confidence intervals.*

**Figure E. Within-family associations between violent victimisation and subsequent common psychiatric disorders, suicidal behaviours, and premature mortality in Finland and Sweden with varying washout periods (e.g., 1 month, 6 months, 12 months, and 24 months)**


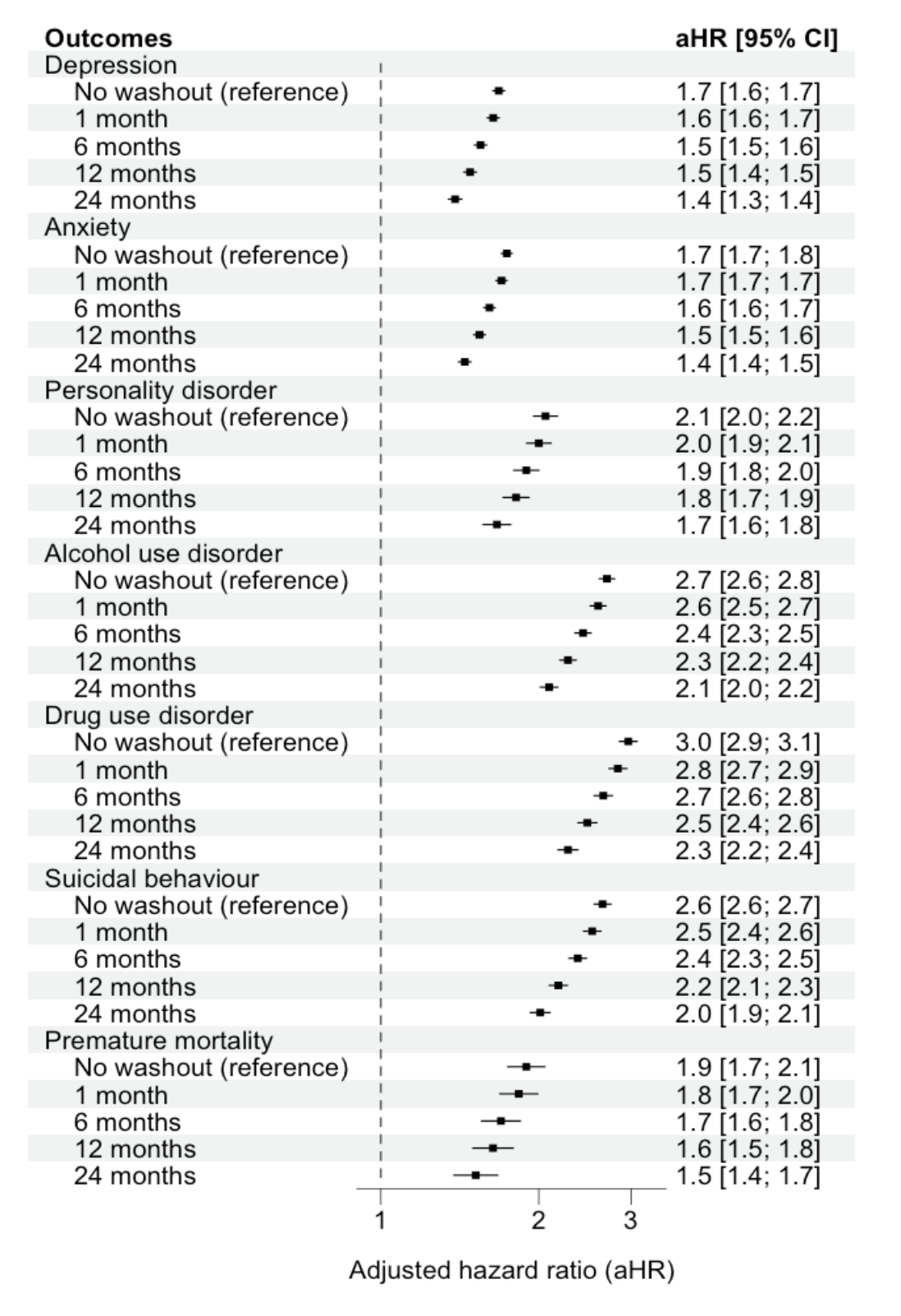


*Notes: The estimates refer to within-family associations comparing biological full-siblings differentially exposed to violent victimisation and is adjusted for all time-invariant unmeasured familial confounders shared between the siblings as well as the following measured confounders that vary within families: sex, birth year, birth order, and parental characteristics at birth (low family income, single mother, psychiatric history, and violent crime history) as well as any pre-existing psychiatric disorders and self-harm events. CI refers to confidence intervals.*

**Figure F. Within-family associations between violent victimisation and subsequent common psychiatric disorders, suicidal behaviours, and premature mortality in Finland and Sweden and adjusted for additional indicators for parental socioeconomic status (SES)**

**
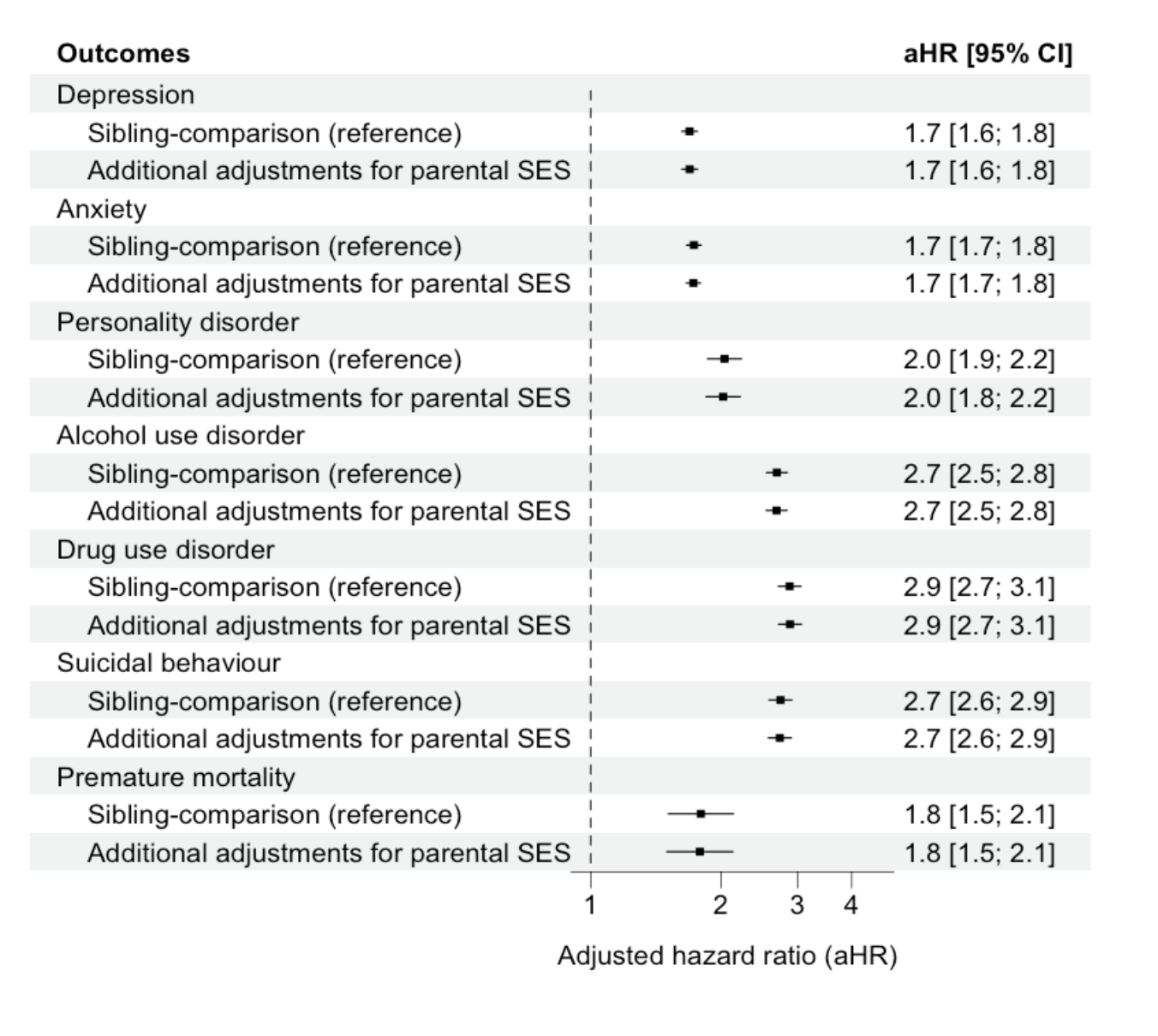
**

*Notes: The additional indicators include parental educational attainment, unemployment status, disability pension and social assistance benefits measured at offspring birth. The estimates refer to within-family associations comparing biological full-siblings differentially exposed to violent victimisation and is adjusted for all time-invariant unmeasured familial confounders shared between the siblings as well as the following measured confounders that vary within families: sex, birth year, birth order, and parental characteristics at birth (low family income, single mother, psychiatric history, and violent crime history) as well as any pre-existing psychiatric disorders and self-harm events. CI refers to confidence intervals.*

**Figure G. Within-family associations between violent victimisation and subsequent common psychiatric disorders and suicidal behaviours (at least two diagnoses on separate occasions) in Finland and Sweden**

**
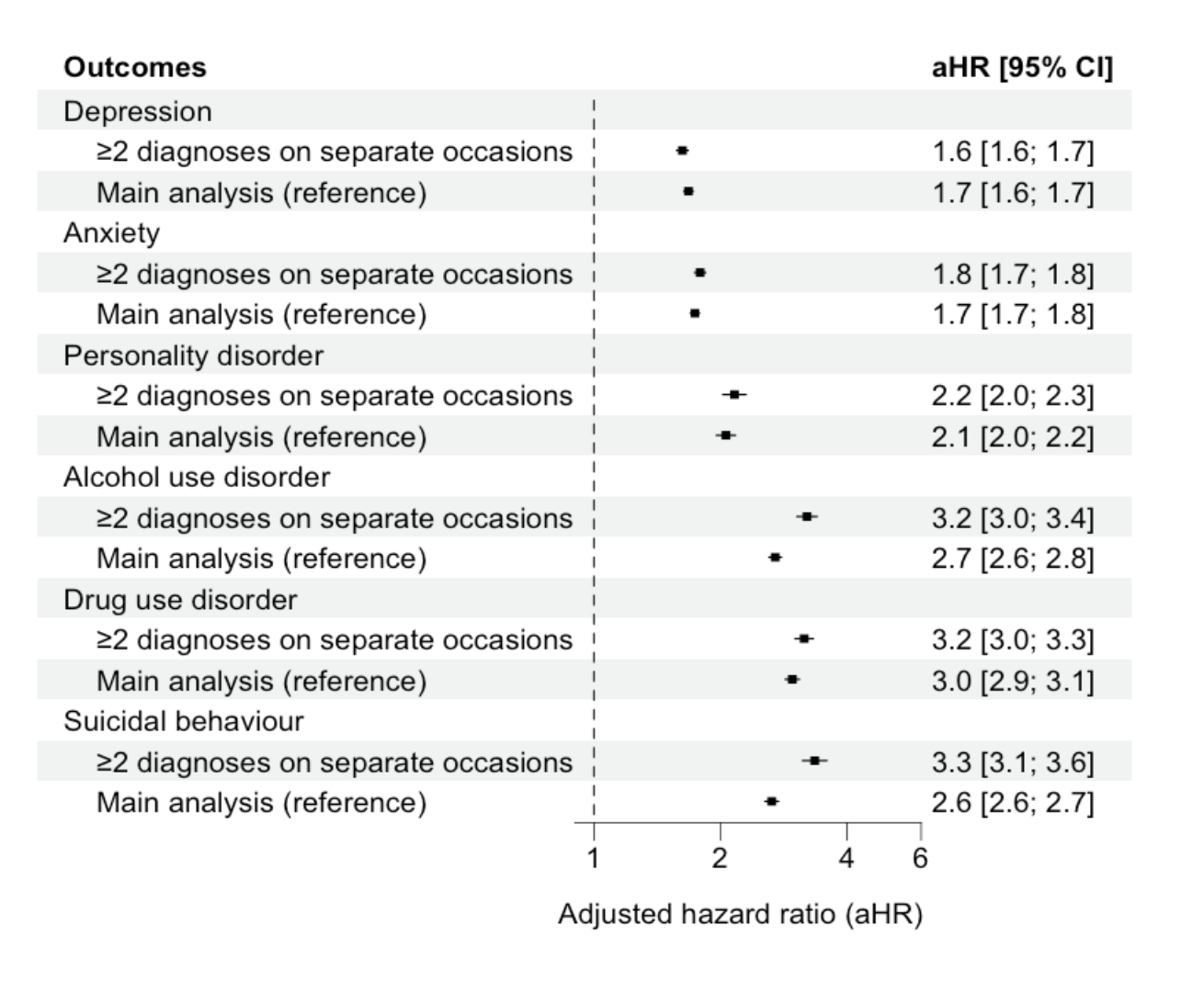
**

*Notes: The estimates refer to within-family associations comparing biological full-siblings differentially exposed to violent victimisation and is adjusted for all time-invariant unmeasured familial confounders shared between the siblings as well as the following measured confounders that vary within families: sex, birth year, birth order, and parental characteristics at birth (low family income, single mother, psychiatric history, and violent crime history) as well as any pre-existing psychiatric disorders and self-harm events. CI refers to confidence intervals.*

**Figure H. Within-family associations between violent victimisation and subsequent common psychiatric disorders, suicidal behaviours, and premature mortality in Finland and Sweden stratified across the entire period and the most recent period (2006-2020)**

**
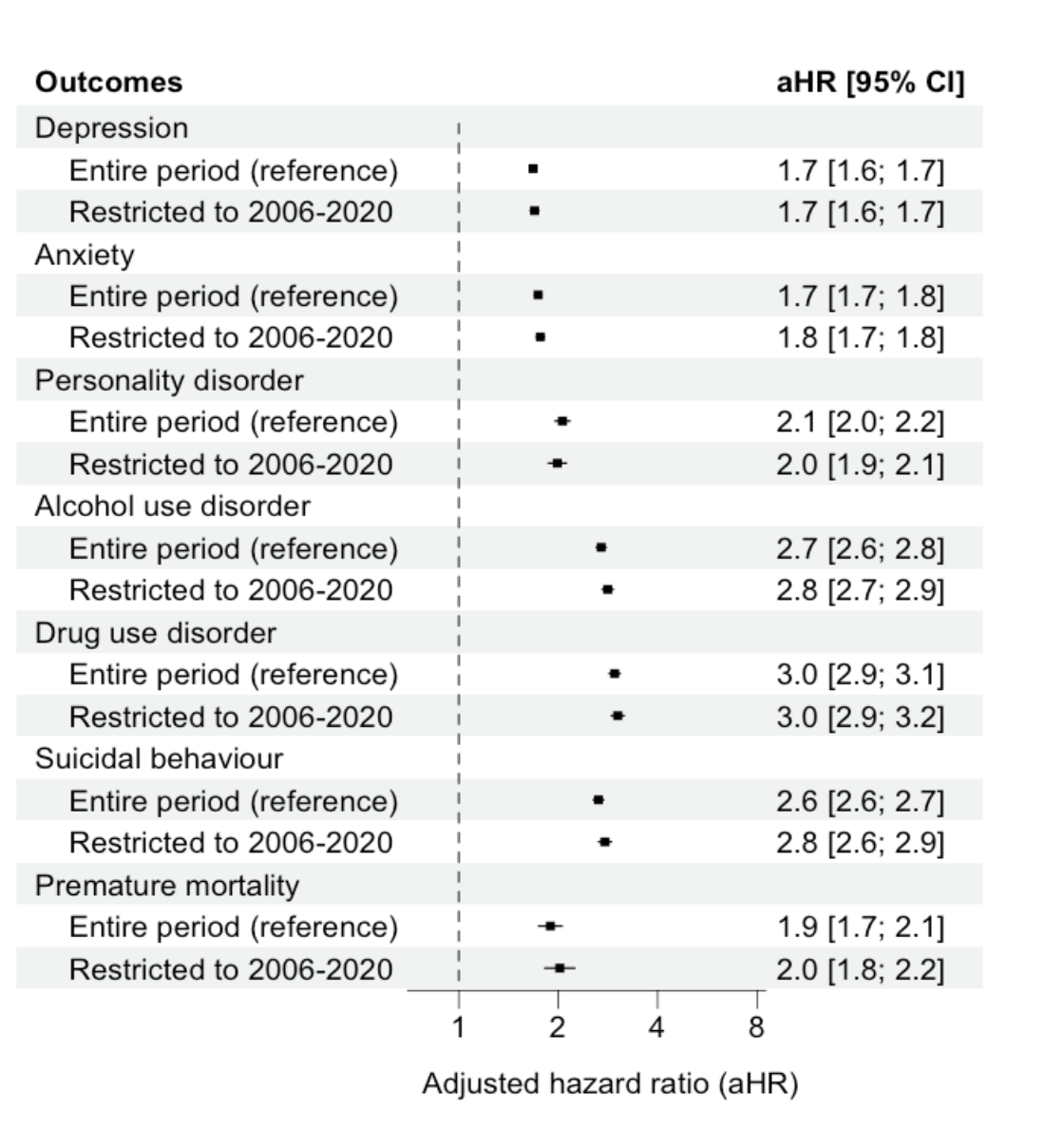
**

*Notes: The estimates refer to within-family associations comparing biological full-siblings differentially exposed to violent victimisation and is adjusted for all time-invariant unmeasured familial confounders shared between the siblings as well as the following measured confounders that vary within families: sex, birth year, birth order, and parental characteristics at birth (low family income, single mother, psychiatric history, and violent crime history) as well as any pre-existing psychiatric disorders and self-harm events. CI refers to confidence intervals.*

**Figure I. Within-family associations between violent victimisation and subsequent common psychiatric disorders, suicidal behaviours, and premature mortality in Finland and Sweden, excluding individuals with pre-existing psychiatric disorders**


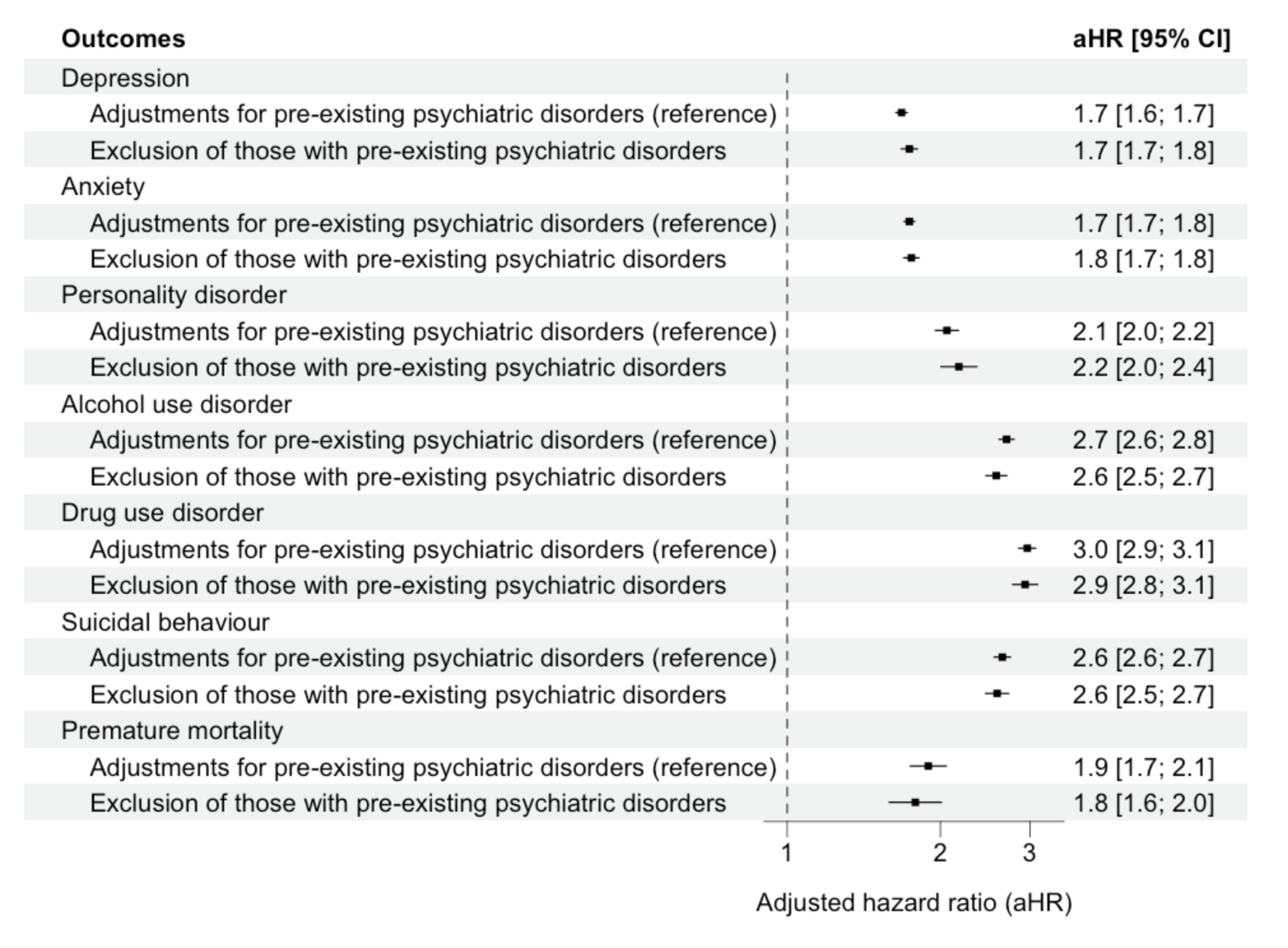


*Notes: The estimates refer to within-family associations comparing biological full-siblings differentially exposed to violent victimisation and is adjusted for all time-invariant unmeasured familial confounders shared between the siblings as well as the following measured confounders that vary within families: sex, birth year, birth order, and parental characteristics at birth (low family income, single mother, psychiatric history, and violent crime history). CI refers to confidence intervals.*

**Figure J. Population-wide and within-family associations between violent victimisation and subsequent stress-related disorders in Finland and Sweden**


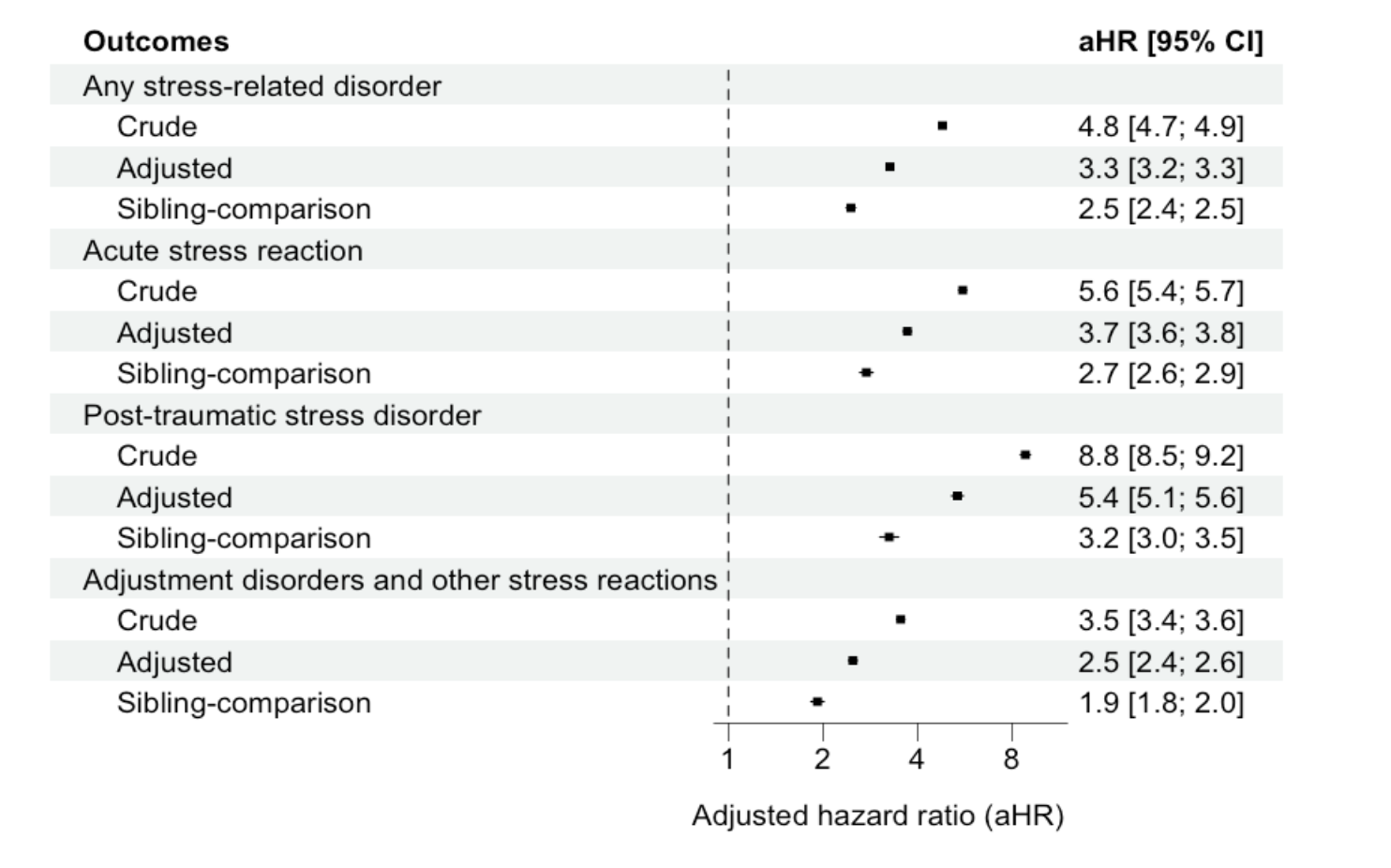


*Notes: The ‘Crude’ model adjusted for sex and birth year. The ‘Adjusted’ model additionally accounted for birth order, parental immigrant background, low family income at offspring birth, single mother at offspring birth, parental psychiatric and violent crime histories at offspring birth, as well as pre-victimisation psychiatric disorders, substance use disorders and self-harm. The ‘Sibling-comparison’ model refers to within-family estimates comparing biological full-siblings differentially exposed to violent victimisation and is adjusted for all time-invariant unmeasured familial confounders shared between the siblings as well as the following measured confounders that vary within families: sex, birth year, birth order, and parental characteristics at birth (low family income, single mother, psychiatric history, and violent crime history) as well as any pre-existing psychiatric disorders and self-harm events. CI refers to confidence intervals.*
